# An integrative analysis of the transcriptome and proteome of the pulp of a spontaneous late-ripening sweet orange mutant and its wild type improves our understanding of fruit ripening in citrus

**DOI:** 10.1093/jxb/eru044

**Published:** 2014-03-05

**Authors:** Juxun Wu, Zhilong Xu, Yajian Zhang, Lijun Chai, Hualin Yi, Xiuxin Deng

**Affiliations:** ^1^Key Laboratory of Horticultural Plant Biology, Ministry of Education, Huazhong Agricultural University, Wuhan 430070, PR China; ^2^National Key Laboratory of Crop Genetic Improvement, Huazhong Agricultural University, Wuhan 430070, PR China

**Keywords:** Abscisic acid, ethylene, fruit ripening, iTRAQ, proteome, sucrose, transcriptome.

## Abstract

Fruit ripening is a complex, genetically programmed process that occurs in conjunction with the differentiation of chloroplasts into chromoplasts and involves changes to the organoleptic properties of the fruit. In this study, an integrative analysis of the transcriptome and proteome was performed to identify important regulators and pathways involved in fruit ripening in a spontaneous late-ripening mutant (‘Fengwan’ orange, *Citrus sinensis* L. Osbeck) and its wild type (‘Fengjie 72-1’). At the transcript level, 628 genes showed a 2-fold or more expression difference between the mutant and wild type as detected by an RNA sequencing approach. At the protein level, 130 proteins differed by 1.5-fold or more in their relative abundance, as indicated by iTRAQ (isobaric tags for relative and absolute quantitation) analysis. A comparison of the transcriptome and proteome data revealed some aspects of the regulation of metabolism during orange fruit ripening. First, a large number of differential genes were found to belong to the plant hormone pathways and cell-wall-related metabolism. Secondly, we noted a correlation between ripening-associated transcripts and sugar metabolites, which suggests the importance of these metabolic pathways during fruit ripening. Thirdly, a number of genes showed inconsistency between the transcript and protein level, which is indicative of post-transcriptional events. These results reveal multiple ripening-associated events during citrus ripening and provide new insights into the molecular mechanisms underlying citrus ripening regulatory networks.

## Summary

ABA, ethylene, sucrose, and their related genes and pathways are involved in fruit ripening of citrus, and ABA may play a central role during the ripening process.

## Introduction

Citrus fruits are among the most important and widely grown commodity fruit crops and have a non-climacteric fruit maturation character (Paul *et al.*, 2012). The growth and development of citrus fruits can be split into three stages: cell division, expansion, and ripening (Bain, 1958). At the ripening stage, carotenoids, sugars, and other soluble solids are accumulated, chlorophyll and organic acid contents are reduced, the cell wall is extensively modified, and the concentration of a number of volatiles increases (Katz *et al.*, 2011; Yu *et al.*, 2012).

Plant hormones are very important for fruit development and ripening (Theologis, 1992; Fan *et al.*, 1998; Soto *et al.*, 2013). Recent studies of strawberry (*Fragaria ananassa*) showed that abscisic acid (ABA) promoted fruit ripening and played an important role in the regulation of fruit development and ripening (Ji *et al.*, 2012; Jia *et al.*, 2011, 2013b). Jia *et al.* (2011) applied the VIGS (Tobacco rattle virus-induced gene silencing) technique to silence a 9-*cis*-epoxycarotenoid dioxygenase gene (*FaNCED1*) in strawberry fruit, which is key to ABA biosynthesis, resulting in a significant decrease in ABA levels and uncoloured fruits. Interestingly, a similar uncoloured phenotype was observed in the transgenic RNA interference (RNAi) fruits, in which the expression of a putative ABA receptor gene encoding the magnesium chelatase H subunit (*FaCHLH/ABAR*) was downregulated by virus-induced gene silencing and, more importantly, downregulation of the *FaCHLH/ABAR* gene in the RNAi fruit altered both ABA levels and sugar content as well as a set of ABA- and/or sugar-responsive genes. Koyama *et al.* (2010) found that ABA application rapidly induced the accumulation of anthocyanin and flavonol in berry skins of the Cabernet Sauvignon grape, which proved that ABA could stimulated ripening and ripening-related gene expression. Recently, ABA level was investigated in cucumber (*Cucumis sativus* L.), and a peak in ABA level was found in pulp before the fruit get fully ripe; real-time PCR analysis revealed that the ABA content may be regulated by its biosynthesis (*CsNCEDs*), catabolism (*CsCYP707A1*), and reactivation (*CsBGs*) genes (Wang *et al.*, 2013). Similarly, a study on a spontaneous fruit-specific ABA-deficient mutant sweet orange, ‘Pinalate’, showed that ABA was an important regulator for the onset of fruit degreening and carotenoid biosynthesis (Romero *et al.*, 2012), although other maturation processes were not affected in ‘Pinalate’ fruit (Rodrigo *et al.*, 2003). Rodrigo *et al.*(2006) found that the content of ABA was increased with the fruit-ripening process and that ethylene could stimulate ABA content and promote fruit coloration in orange. Recent studies have indicated that ABA appears to modulate ripening through interference with ethylene and auxin-related genes (Zhang *et al.*, 2009; Ji *et al.*, 2012; Sun *et al.*, 2012; Soto *et al.*, 2013). ABA was shown to have the ability to control the gene expression of the entire system of cell-wall catabolism genes, such as *XET*, *PE*, and *PG* (Sun *et al.*, 2012). These studies suggest that ABA plays an important role in the regulation of fruit ripening. Although ethylene plays a major role in the ripening process of climacteric fruits (Theologis, 1992), studies on citrus, a non-climacteric group of fruits, have shown that ripening-related colour changes in the flavedo portion of the fruit peel are regulated by endogenous as well as exogenous levels of ethylene (Goldschmidt, 1997), and the fruit’s sensitivity to ethylene is also an important factor for fruit ripening; even a small amount of ethylene produced by the fruit might be sufficient to trigger ripening-related physiological responses in some non-climacteric fruits (Perkins-Veazie *et al.*, 1996; Trainotti *et al.*, 2005).

Sugars were traditionally regarded as the metabolic resources required for construction of the carbon skeleton and energy supply in plants. Recently, however, many studies have suggested that sugars may serve as important signals that modulate a wide range of processes in the plant life cycle. Notably, intimate connections between sugar and ABA signalling have been revealed by the isolation and characterization of sugar-insensitive and sugar-hypersensitive mutants in *Arabidopsis* (Smeekens, 2000; Rolland *et al.*, 2006). Other studies have demonstrated that ABA and sugars often have similar or antagonistic effects on diverse developmental processes (Rolland *et al.*, 2006; Jia *et al.*, 2011). Recent work has demonstrated that sucrose is an important signal in the regulation of strawberry fruit ripening (Jia *et al.*, 2013a).

The ripening of citrus fruit is accompanied by the synthesis of a large number of proteins and the transcription of many genes. Several proteins and regulation factors are involved in this process. Transcriptomic and proteomic analyses are extremely efficient methods for identifying differential expression genes at the whole-genome level. Recently, the fruit ripening of the tomato was investigated at the whole-genome level by comparative transcriptomics and proteomics, which highlighted the need for combined transcriptomic and proteomic analyses (Osorio *et al.*, 2011). More specifically, the recently developed isobaric tags for relative and absolute quantitation (iTRAQ) technology was validated to be powerful in protein profiling (Elias and Gygi, 2007; Gan *et al.*, 2007; Noirel *et al.*, 2011; Unwin *et al.*, 2010). However, previous studies revealed that the transcripts did not, in fact, always coincide well with their final products, the proteins (Osorio *et al.*, 2011; Pan *et al.*, 2012). This divergence might be due to post-transcriptional and translational processing that regulates the location, quantity, and efficiency of the proteins in the cell.

In citrus, artificially generated mutants are very difficult to generate, but spontaneous mutants are widely found in nature. A spontaneous late-ripening mutant from the ‘Fengjie 72-1’ orange (*Citrus sinensis* L. Osbeck), named ‘Fengwan’, has been biochemically characterized (Liu *et al.*, 2006b). During natural ripening, the onset of fruit degreening and the time of full maturation were delayed in ‘Fengwan’, compared with its wild-type (WT) cultivar (Liu *et al.*, 2006b). As a result, these two materials, ‘Fengjie 72-1’ and ‘Fengwan’, have provided a promising platform to investigate the regulation network of citrus fruit development and ripening. In the present study, we examined ‘Fengjie 72-1’ and ‘Fengwan’ along the ripening periods at the transcriptomic and proteomic levels. Transcriptional analysis was conducted using RNA sequencing (RNA-seq) (Mortazavi *et al.*, 2008), and proteomic data were obtained using iTRAQ (Elias and Gygi, 2007; Gan *et al.*, 2007; Unwin *et al.*, 2010; Noirel *et al.*, 2011).

## Materials and methods

### Plant materials and RNA preparation

The WT ‘Fengjie 72-1’ orange (*C. sinensis* L. Osbeck) and its spontaneous late-ripening mutant (MT) ‘Fengwan’ were both cultivated in the same orchard (Fengjie, Chongqing City, China). Fruit samples were harvested at 150, 170, 190, 210, and 240 d after flowering (DAF) from three different trees in 2011. Twelve representative fruits were sampled from each tree at each developmental stage. After separating the pulp from the peel, the pulp and peel were sliced. Combining the sliced WT pulp samples with one another (as for the MT samples), the samples were frozen rapidly in liquid nitrogen and kept at –80 °C (Liu *et al.*, 2009; Xu *et al.*, 2009). A portion of the samples was used for extracting the RNA, as described previously (Liu *et al.*, 2006a). Another aliquot was used to extract the protein. A portion of the sample powder was used for the determination of ABA, sugar, and organic acid composition and concentration. Similarly to the sliced pulp samples, the peel samples were frozen rapidly in liquid nitrogen and kept at –80 °C for determination of the total amount of chlorophyll.

### Analysis of total chlorophyll quantification, soluble sugar, and organic acid

Approximately 0.5g of peel material was used for determination of the total chlorophyll content, according to the method described by Li (2000). Each sample was assayed with three replicates. A gas chromatography (GC) method was used to determine the soluble sugar and organic acid composition and the concentrations of the WT and MT fruit pulps harvested at 150, 170, 190, 210, and 240 DAF, as described previously (Yu *et al.*, 2012).

### Quantification of ABA

The samples for ABA quantification were prepared according to the method described by Pan *et al.* (2010) with some modified. D_6_-ABA (Icon Isotopes) was used as an internal standard for ABA. In brief, fruit pulp was ground into powder with a mortar and pestle in liquid nitrogen, weighed and each sample (200mg) was transferred to 10ml screw-cap tubes. The samples were kept in liquid nitrogen. Fifty microliters of the working solution of internal standards was added to each 10ml tube containing the frozen plant material and 2ml of extraction solvent [2-propanol:H_2_O:concentrated HCl (2:1:0.002, v/v/v)], was added to each tube. The tubes were placed on a shaker at a speed of 200rpm for 30min at 4 °C. Dichloromethane (4ml) was added to each sample and shaken for 30min at 4 °C. The samples were placed into a refrigerated microcentrifuge at 4 °C and centrifuged at 13 000*g* for 5min. Approximately 4ml of the solvent was transferred from the lower phase into a screw-cap vial and the solvent mixture was concentrated (not completely dry) using a nitrogen evaporator with nitrogen flow. The samples were redissolved in 0.2ml of methanol and filtered with 0.22 μm organic membrane filters. The extracted solution was injected into a reverse-phase C_18_ Gemini high-performance liquid chromatography (HPLC) column for HPLC electrospray ionization tandem mass spectrometry (HPLC-ESI-MS/MS) analysis. An Agilent 1100 HPLC (Agilent Technologies), Waters C_18_ column (150×2.1mm, 5 µm), and API3000 MS-MRM (Applied Biosystems) were used as for the analysis. The reaction monitoring conditions (Q1/Q3) of ABA and D_6_-ABA were 263.00/153.00 and 269.00/159.10, respectively. Each sample was assayed using four replicates.

### RNA-seq, data processing, and gene annotation

**Fig. 1.**
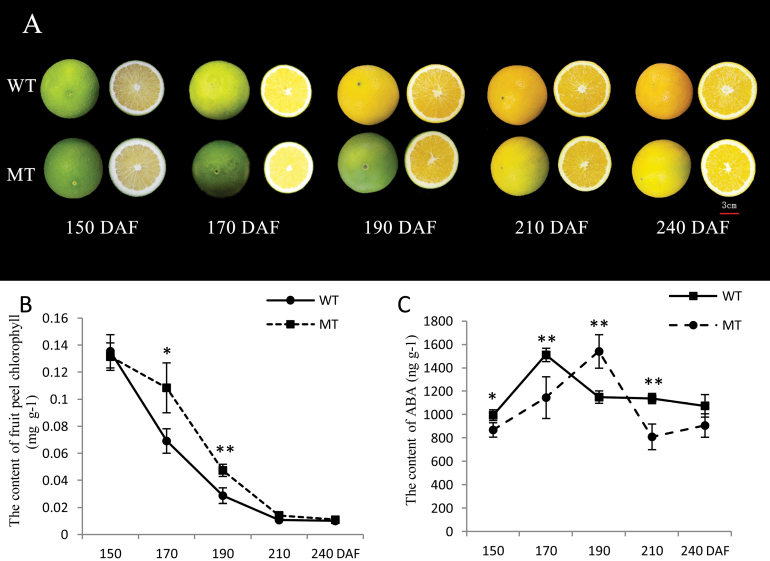
(A) Phenotypic characterization of fruit development in MT and WT. (B) Chlorophyll content in the fruit peel of MT and WT at five developmental stages. (C) ABA content in the fruit pulp of MT and WT at five developmental stages. Bars represent the standard error (SE; *n*=3). Asterisks represents statistically significant differences (*P<0.05; **P<0.01) analysed using Student’s *t*-test. (This figure is available in colour at *JXB* online.)

**Fig. 2.**
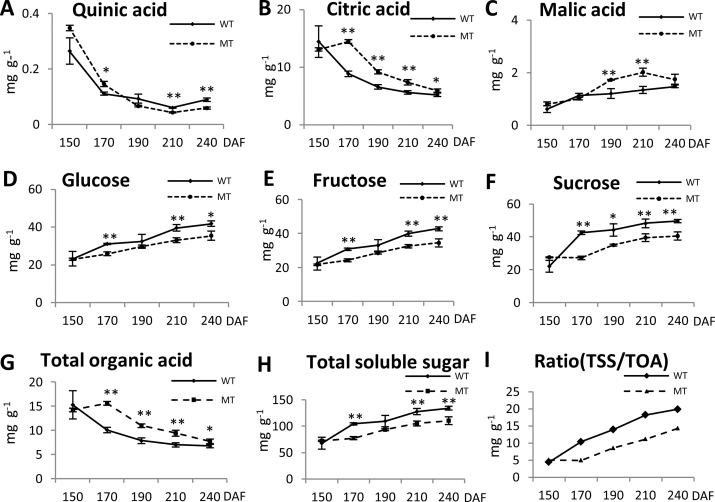
Trends in organic acid and soluble sugar content in MT and WT fruit pulp (fresh weight) during fruit ripening. Quinic acid (A), citric acid (B), malic acid (C), glucose (D), fructose (E), and sucrose (F) content were extracted at 150, 170, 190, 210, and 240 DAF. Three individual replicates were used to reduce the experimental error. The total organic acid (TOA; G) and total soluble sugar (TSS; H) were the sum of the organic acids (malic acid, citric acid, and quinic acid) and the soluble sugars (fructose, glucose, and sucrose), respectively. The ratio of TSS:TOA was determined (I). Bars represent the SE (*n*=3). Asterisks represents statistically significant differences (*P<0.05; **P<0.01) analysed using Student’s *t*-test.

Combining the data of ABA, chlorophyll, soluble sugar, and organic acid (Figs 1 and 2), We found that the increase or decrease in sugars and acids and the largest peel colour change of fruit, as well as the ABA peak, all appeared at 170, 190, and 210 DAF, indicating that these three ripening stages were the key stages in fruit ripening. Thus, the WT and MT fruit pulps harvested at 170, 190, and 210 DAF were subjected to RNA-seq using an Illumina HiSeq^TM^2000 at the Beijing Genomics Institute (Shenzhen) in 2011. Briefly, 6 μg of the total RNA of each sample was used to enrich the mRNA, to construct cDNA libraries, and for sequencing analysis. High-quality reads (clean reads) were obtained by removing low-quality reads with ambiguous nucleotides, and adaptor sequences were filtered from the raw reads, and a statistical analysis was conducted to summarize the number of clean reads that aligned with the recently released reference genome (Xu *et al.*, 2013); gene expression levels were calculated by the RPKM (reads per kb per million reads) method according to Zheng *et al.* (2012).

Gene annotation was conducted using the Blast2GO program. Gene ontology (GO) enrichment analysis provided all of the GO terms that were significantly enriched in differentially expressed genes (DEGs) compared with the genome background, and we filtered the DEGs that corresponded to biological functions. The biological interpretation of the differential genes was further completed by assigning them to metabolic pathways using the Kyoto Encyclopaedia of Genes and Genomes (KEGG) annotation.

### Sequence alignment and real-time quantitative PCR verification

Sequence similarity comparisons between *Arabidopsis thaliana* and *C. sinensis* proteins were performed by BLASTP in the *Citrus sinensis* Annotation Project (CAP) (http://citrus.hzau.edu.cn/). A search for amino acid sequences of *Arabidopsis* ABA- and ethylene-related proteins was carried out using the National Centre for Biotechnology Information (Supplementary Table S1 available at *JXB* online).

A total of 1 μg of RNA was reverse transcribed for first-strand cDNA synthesis using a RevertAid^TM^ First Strand cDNA synthesis kit (Fermentas) according to the manufacturer’s instructions. The gene-specific primer pairs (Supplementary Tables S1 and S2 available at *JXB* online), which were designed with the Primer Express 3.0 software (Applied Biosystems), were used for real-time PCR. Reactions were performed with the SYBR Green PCR Master Mix in an ABI 7900HT Fast Real-time system. *Actin* was used as the standard to normalize the content of cDNA as described previously (Liu *et al.*, 2009). Ten microlitres of the reaction mixture was added to each well. The thermal cycling program was set at 50 °C for 2min, 95 °C for 1min, and 40 cycles of 95 °C for 15 s and 60 °C for 1min. The output results were analysed by the instrument on-board software Sequence Detector Version 1.3.1 (PE Applied Biosystems). The real-time PCR was conducted with five replicates for each sample, and data are indicated as means ±standard error (SE) (*n*=3).

### Protein extraction, protein digestion, and iTRAQ labelling

The WT and MT fruit pulps harvested at 170, 190, and 210 DAF were used for protein extraction, which was performed using phenol extraction, as described previously (Pan *et al.*, 2009). The protein concentration was determined with a Bio-Rad Protein Assay kit based on the Bradford method using BSA as a standard. Two independent protein extractions were performed.

One hundred micrograms of total protein from each sample solution was used for protein digestion. First, the protein was digested with Trypsin Gold at a protein:trypsin ratio of 20:1 (w/w) at 37 °C for 4h. The Trypsin Gold was then added at a protein:trypsin ratio of 20:1 (w/w) once more, and the result was digested for 8h continuously.

After trypsin digestion, the peptide was dried by vacuum centrifugation. The peptide was reconstituted in 0.5M triethylammonium bicarbonate buffer and processed according to the manufacturer’s protocol for 8-plex iTRAQ (Applied Biosystems). Briefly, 1U of iTRAQ reagent (defined as the amount of reagent required to label 100 μg of protein) was thawed and reconstituted in 70 μl of isopropanol. Peptides from digestion were labelled with different iTRAQ tags in the same group by incubation at room temperature for 2h. Samples of WT fruit pulp harvested at 170, 190, and 210 DAF were each labelled with iTRAQ reagents with molecular masses of 114, 116, and 119Da, respectively. Samples of MT fruit pulp harvested at 170, 190, and 210 DAF were each labelled with iTRAQ reagents with molecular masses of 113, 115, and 118Da, respectively. After labelling, the peptide mixtures were pooled and dried by vacuum centrifugation. The pooled mixtures of iTRAQ-labelled peptides were fractionated by strong cationic exchange (SCX) chromatography.

### Fractionation by SCX

For SCX chromatography using a Shimadzu LC-20AB HPLC Pump system, the iTRAQ-labelled peptides were reconstituted with 4ml of buffer A (25mM NaH_2_PO_4_ in 25% acetonitrile, pH 2.7) and loaded onto a 4.6×250mm Ultremex SCX column that contained 5 μm particles (Phenomenex). The peptides were eluted at a flow rate of 1ml min^–1^ with a gradient of buffer A for 10min, 5–35% buffer B (25mM NaH_2_PO_4_, 1M KCl in 25% acetonitrile, pH 2.7) for 11min and 35–80% buffer B for 1min. The system was then maintained in 80% buffer B for 3min before equilibrating with buffer A for 10min prior to the next injection. Elution was monitored by measuring the absorbance at 214nm, and the fractions were collected every 1min. The eluted peptides were pooled as 12 fractions, desalted using a Strata X C18 column (Phenomenex), and vacuum dried.

### LC-ESI-MS/MS analysis by LTQ Orbitrap HCD

Each fraction was resuspended in a certain volume of buffer A (2% acetonitrile, 0.1% formic acid) and centrifuged at 20 000*g* for 10min. In each fraction, the final concentration of peptide was approximately 0.5 μg μl^–1^, on average. A total of 10 μl of supernatant was loaded onto a Shimadzu LC-20AD nanoHPLC by the autosampler onto a 2cm C18 trap column (inner diameter, 200 μm), and the peptides were eluted onto a resolving 10cm analytical C18 column (inner diameter, 75 μm) made in house. The samples were loaded at 15 μl min^–1^ for 4min; the 44min gradient was then run at 400 nl min^–1^ from 2 to 35% B (98% acetonitrile, 0.1% formic acid), followed by a 2min linear gradient to 80%, maintenance at 80% B for 4min, and finally a return to 2% for 1min.

The peptides were subjected to nanoESI followed by MS/MS in an LTQ Orbitrap Velos (Thermo Scientific) that was coupled online to the HPLC. Intact peptides were detected in the Orbitrap at a resolution of 60 000. Peptides were selected for MS/MS using a high-energy collision dissociation (HCD) operating mode with a normalized collision energy setting of 45%; the ion fragments were detected in the LTQ. A data-dependent procedure that alternated between one MS scan followed by eight MS/MS scans was applied for the eight most abundant precursor ions above a threshold ion count of 5000 in the MS survey scan with the following Dynamic Exclusion settings: repeat counts, 2; repeat duration, 30 s; and exclusion duration, 120 s. The electrospray voltage applied was 1.5kV. Automatic gain control was used to prevent overfilling of the ion trap; 1×10^4^ ions were accumulated in the ion trap for the generation of the HCD spectra. For the MS scans, the *m*/*z* scan range was 350–2000Da.

### Database search and quantification

Mascot software version 2.3.02 (Matrix Science) was used to simultaneously identify and quantify the proteins. In this version, only unique peptides used for protein quantification can be chosen, which provides a more precise quantification of the proteins. Searches were made against the database (44272 sequences; http://citrus.hzau.edu.cn/orange/) (Xu *et al.*, 2013). Spectra from the 12 fractions were combined into one MGF (Mascot generic format) file after loading the raw data, and the MGF file was searched. The search parameters were as follows: trypsin was chosen as the enzyme with one missed cleavage allowed; fixed modifications of carbamidomethylation at Cys, variable modifications of oxidation at Met; peptide tolerance was set at 0.05Da, and MS/MS tolerance was set at 0.1Da. The peptide charge was set to ‘Mr’, and the monoisotopic mass was chosen. An automatic decoy database search strategy was also employed to estimate the false discovery rate (FDR); the FDR was calculated as the number of false-positive matches divided by the total number of matches. In the final search results, the FDR was less than 1.5%. The iTRAQ 8-plex was chosen for quantification during the search.

The search results were passed through additional filters before exporting the data. For protein identification, the filters were set as follows: significance threshold *P*<0.05 (with 95% confidence) and an ion score or expected cut-off of less than 0.05 (with 95% confidence). For protein quantitation, the filters were set as follows: ‘median’ was chosen for the protein ratio type (http://www.matrixscience.com/help/quant_config_help.html); the minimum precursor charge was set to 2+, and the minimum peptide was set to 2; only unique peptides were used to quantify the proteins. The median intensities were set to normalization, and outliers were removed automatically. The peptide threshold was set as above for identity.

## Results

### Changes in soluble sugars, organic acid, and chlorophyll content during fruit ripening in MT and WT

Fruit taste is determined by the content and types of soluble sugars and organic acids. Thus, the levels of soluble sugars and organic acids of MT and WT were determined during fruit ripening. In ripe citrus fruit, the main soluble sugars are sucrose, fructose, and glucose. The trends for organic acid and soluble sugar content are shown in Fig. 2. The content of the soluble sugars increased markedly during the stages of fruit ripening in both WT and MT (Fig. 2D–F, H). The content of the soluble sugars in MT were all obviously lower than that in WT from 170 to 240 DAF (Fig. 2D–F). In contrast, the concentrations of citric acid, quinic acid, and total organic acid (TOA) decreased with the development of fruit ripening in both WT and MT (Fig. 2A, B, G). However, the malic acid concentrations increased during the stages of fruit ripening (Fig. 2C). In addition, we observed that the concentrations of citric acid, malic acid, and TOA were higher in MT compared with WT from 170 to 240 DAF, but the concentrations of quinic acid were lower. Thus, the decrease in TOA, citric acid, and quinic acid in MT and WT fruit and the increase in total soluble sugar (TSS), malic acid, and sucrose, and the change in the ratio of TSS:TOA were significant after 170 DAF. The chlorophyll content of the WT and MT peels were also determined at five stages (Fig. 1B). We observed that, at stages following mature green (170 DAF), the mutant fruit diverged in its development from the WT, as the mutant is late ripening (Fig. 1A).This finding suggested that 170 DAF was the turning point at which the MT fruit diverged in its development from the WT.

### Fruit transcriptome difference between MT and WT during fruit ripening

**Fig. 3.**
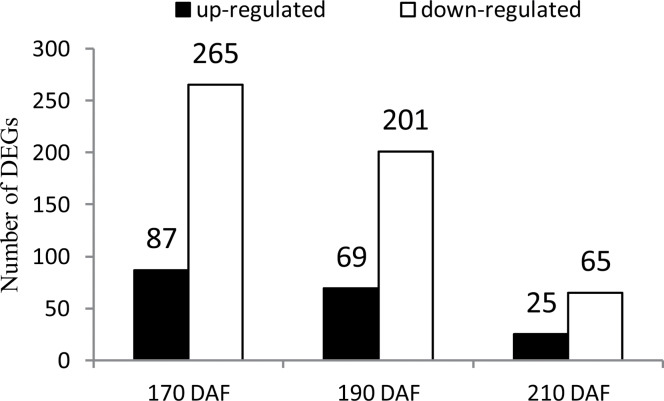
Number of DEGs between MT and WT at 170, 190, and 210 DAF.

One of the primary goals of transcriptome sequencing (Supplementary Results available at *JXB* online) is to compare the gene expression levels in two genotypes. In this study, we used a stringent value of FDR ≤0.001 and a *P* value <0.05 as the threshold to judge the significant differences in the gene expressions. A total of 628 genes were differently expressed (≥2-fold) between MT and WT (Supplementary Table S3 available at *JXB* online); of these, 352 were at 170 DAF, 270 were at 190 DAF, and 90 were at 210 DAF (Supplementary Table S4 available at *JXB* online). At all three developmental stages, the number of upregulated genes was less than the number of downregulated genes, and the number of DEGs was decreased following fruit development (Fig. 3).

**Fig. 4.**
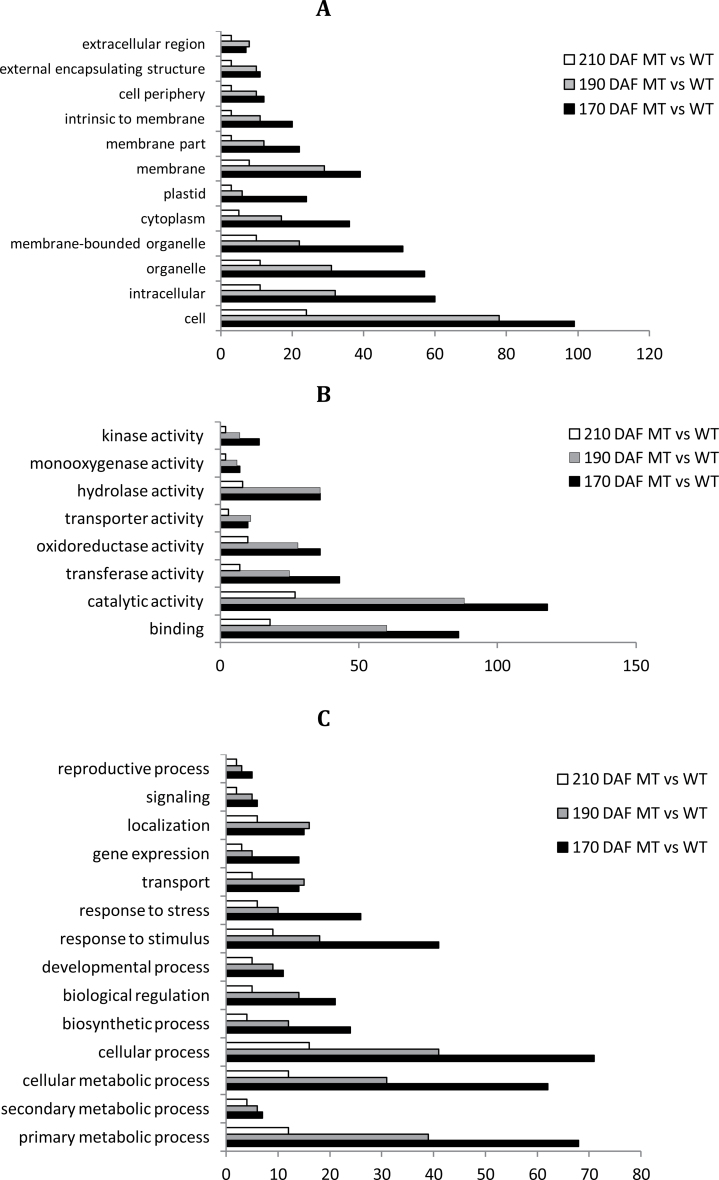
Functional categorization of genes with significant transcriptional differences between MT and WT. The genes were categorized based on GO annotation, and the number of each category is displayed based on cellular components (A), molecular function (B), or biological process (C).

To gain insight into the functional categories that were altered between MT and WT, GO categories were assigned to the 628 DEGs with a BLASTX hit using Blast2GO. Fig. 4 shows the distributions of the GO terms according to the corresponding biological process, molecular function, and cellular component. Cell (99 genes distributed in 170 DAF, 78 genes distributed in 190 DAF, and 24 genes distributed in 210 DAF), intracellular (60, 32, and 11), organelle (57, 31, and 11) and membrane (39, 29, and 8) were the major categories annotated for the cellular component (Fig. 4A). Binding (86, 60, and 18), catalytic activity (118, 88, and 27), hydrolase activity (36, 36, and 8), transferase activity (43, 25, and 7), and oxidoreductase activity (36, 28, and 10) were the major categories annotated under molecular function (Fig. 4B). According to the GO terms of the biological process, the majority of DEGs appeared to be related to four major biological changes, including the primary metabolic process (68, 39, and 12), cellular process (71, 41, and 16), cellular metabolic process (62, 31, and 12), and response to stimulus (41, 18, and 9) (Fig. 4C). In addition, we noted that the number of DEGs distributed to the secondary metabolic processes (7, 6, and 4) was much less than the number distributed to the primary metabolic processes (68, 39, and 12) (Fig. 4C). KEGG analysis assigned the differential genes to 54 metabolic pathways (each of which contained three or more differential genes). The complete list of metabolic pathways is provided in Supplementary Table S5 available at *JXB* online. Table 1 lists the metabolic/biological pathways that contained over five differential genes. Notably, 16 differential genes were predicted to be involved in plant hormone signal transduction at 170 DAF. Similar to previous studies (Zhang *et al.*, 2009; Sun *et al.*, 2012; Jia *et al.*, 2013a), the metabolic pathways of plant hormone signal transduction, carotenoid biosynthesis, flavonoid biosynthesis, and starch and sucrose metabolism were involved in fruit ripening (Table 1).

**Table 1.** KEGG pathways of more than five differential genes in MT compared with WT at 170, 190, and 210 DAF

KEGG pathway	Gene number		
	170 DAF	190 DAF	210 DAF
Plant hormone signal transduction	16	11	1
Phenylpropanoid biosynthesis	15	13	5
Microbial metabolism in diverse environments	15	9	4
Stilbenoid, diarylheptanoid, and gingerol biosynthesis	14	8	4
Plant-pathogen interaction	13	14	2
Protein processing in endoplasmic reticulum	12	1	–
Flavonoid biosynthesis	11	7	4
Polycyclic aromatic hydrocarbon degradation	10	6	3
Starch and sucrose metabolism	10	7	2
Limonene and pinene degradation	9	6	3
Aminobenzoate degradation	9	7	3
Pentose and glucuronate interconversions	8	3	1
Carotenoid biosynthesis	6	–	2
Glycine, serine and threonine metabolism	6	2	2
Phenylalanine metabolism	6	8	1
Antigen processing and presentation	5	1	–
Cyanoamino acid metabolism	5	2	–
Apoptosis	5	3	–
Diterpenoid biosynthesis	4	5	1
Methane metabolism	4	5	2
Lysosome	3	5	3
Glycerophospholipid metabolism	3	5	3
RNA transport	1	5	1

Plant hormones are very important for fruit development and ripening. In this study, as shown in Table 2, 11 genes were found to be involved in plant hormone metabolism, including five genes involved in ABA synthesis and signal transduction (Cs5g14370, orange1.1t03687, Cs1g09250, Cs6g19380, and Cs8g13770) and six genes related to ethylene (Cs2g02500, Cs4g13870, Cs5g29870, Cs9g08850, Cs4g17960, and Cs8g12880), and most of these genes were downregulated in MT. Notably, some key enzymes for synthesizing plant hormones were found among these 11 genes, such as *NCED1* (Cs5g14370), *ACO* (Cs2g02500, Cs4g13870), *AAO* (Cs8g13770), and ABA8′-hydroxylase (orange1.1t03687, Cs1g09250, and Cs6g19380) (Table 2). Some important regulators involved in ethylene signal transduction were identified, including *ETR* (Cs9g08850, Cs8g12880), *EBF4* (Cs4g17960), and *ERF1B* (Cs5g29870) (Table 2). In addition, 15 genes were identified to be involved in cell-wall metabolism, including five *XET*s, five *PE*s, two *PG*s, and three other genes (Table 2). The five *XET*s displayed a mixed expression pattern (two genes upregulated and three genes downregulated), and β-glucosidase also displayed a mixed expression pattern (Table 2). However, the five *PE*s were all downregulated in MT. In contrast, the two *PG*s were all upregulated in MT. The three sucrose-related genes were identified as sucrose synthase (Cs6g15930), sucrose-phosphate synthase (Cs5g19060), and citrus sucrose transporter 1 (Cs3g22560); these genes were all downregulated in MT (Table 2).

**Table 2.** A list of some important genes that are differentially expressed between MT and WT, and are involved in plant hormone biosynthesis/signal transduction, cell-wall metabolism and sucrose-related genesThe BLAST annotation includes the gi number, e-value, gene description and species for the gene.

Gene ID	log_2_ ratio (MT/WT)			BLAST annotation
	170 DAF	190 DAF	210 DAF	
Plant hormone biosynthesis and signal transduction				
Cs5g14370	–2.21	–	–	gi|68300870/0/9-cis-epoxycarotenoid dioxygenase 1 [*Citrus sinensis*]
orange1.1t03687	–2.12	–	–	gi|255544242/1.64691e-52/ABA 8′-hydroxylase [*Ricinus communis*]
Cs1g09250	–1.52	–	–	gi|225445688/1.75384e-79/ ABA 8′-hydroxylase [*Vitis vinifera*]
Cs6g19380	–1.07	–	–	gi|367465454/0/ABA 8′-hydroxylase [*Citrus sinensis*]
Cs8g13770	–	–	–1.08	gi|147841197/0/abscisic-aldehyde oxidase [*Vitis vinifera*]
Cs2g02500	–1.18	–	–	gi|642062/2.15051e-87/1-aminocyclopropane-1-carboxylate oxidase [*Pelargonium*×*hortorum*]
Cs4g13870	–	–1.74	–2.27	gi|359484312/3.38653e-128/PREDICTED: 1-aminocyclopropane-1-carboxylate oxidase 1 [*Vitis vinifera*]
Cs5g29870	–2.47	–	–	gi|224082796/2.90568e-45/ Ethylene-responsive transcription factor 1B [*Populus trichocarpa*]
Cs9g08850	–2.12	–	–	gi|283520944/0/ethylene response 2 [*Citrus sinensis*]
Cs4g17960	–1.94	–	–	gi|224090409/0/ein3-binding f-box protein 4 [*Populus trichocarpa*]
Cs8g12880	–1.13	–	–	gi|283520946/0/ethylene response 3 [*Citrus sinensis*]
Cell-wall metabolism				
Cs2g14920	–1.93	–	–	gi|1008904/9.98188e-140/xyloglucan endotransglycosylase [*Tropaeolum majus*]
Cs8g03550	–1.62	–	–	gi|356534254/1.10015e-133/xyloglucan endotransglucosylase/hydrolase protein 10-like [*Glycine max*]
Cs8g15720	–1.14	–2.52	–	gi|156739650/3.09167e-61/xyloglucan endotraglucosylase/hydrolase [*Vigna angularis*]
Cs4g03220	–	1.57	–	gi|225446121/3.36075e-123/xyloglucan endotransglucosylase/hydrolase protein 23 [*Vitis vinifera*]
Cs1g21130	–	1.29	–	gi|308229788/3.32813e-94/ xyloglucan endotransglucosylase/hydrolase [*Gossypium hirsutum*]
Cs7g19180	–2.23	–	–	gi|356499962/1.10841e-105/pectinesterase PPME1-like [*Glycine max*]
Cs4g15560	–1.88	–	–	gi|255546301/8.86701e-60/Pectinesterase PPE8B precursor [*Ricinus communis*]
Cs9g14330	–1.76	–	–1.13	gi|255539737/3.46069e-48/pectinesterase [*Ricinus communis*]
Cs5g09370	–1.03	–	–	gi|255537037/9.95994e-65/pectinesterase [*Ricinus communis*]
Cs3g14650	–	–10.48	–	gi|224123034/7.78899e-118/pectinesterase [*Populus trichocarpa*]
Cs9g14590	–2.91	–	–	gi|115548295/0/beta-fructofuranosidase [*Citrus sinensis*]
Cs9g01630	1.41	1.56	–	gi|224101497/9.34362e-81/predicted protein [*Populus trichocarpa*]
Cs7g08820	1.08	1.03	–	gi|255568780/2.94863e-76/polygalacturonase, putative [*Ricinus communis*]
Cs8g03370	1.45	1.24	–	gi|356523324/1.43914e-50/PREDICTED: beta-glucosidase 11-like [*Glycine max*]
Cs8g08680	–	–1.16	–	gi|255573163/0/Beta-glucosidase, putative [*Ricinus communis*]
Sucrose-related genes				
Cs6g15930	–	–2.67	–	gi|357123064/0/ sucrose synthase 2-like [*Brachypodium distachyon*]
Cs5g19060	–	–1.97	–	gi|255561468/1.91935e-167/sucrose-phosphate syntase, putative [*Ricinus communis*]
Cs3g22560	–	1.42	–	gi|21063921/0/citrus sucrose transporter 1 [*Citrus sinensis*]

To obtain a global overview of the genes involved in fruit ripening, we summarized the change in the gene expression levels during fruit ripening for WT and MT (Supplementary Tables S7 and S8 available at *JXB* online). We observed that there were 1036 developmentally DEGs in WT and 1406 in MT. KEGG analysis assigned the DEGs of WT and MT to different metabolic pathways. Supplementary Table S6 available at *JXB* online lists some important metabolic/biologic pathways. Notably, most of the DEGs of WT and MT were annotated to ‘Plant–pathogen interaction’, ‘Plant hormone signal transduction’, ‘Stilbenoid, diarylheptanoid and gingerol biosynthesis’, ‘Microbial metabolism in diverse environments’, and ‘Phenylpropanoid biosynthesis’ (Supplementary Table S6 available at *JXB* online).

Some of the important DEGs are listed in Table 3. Several DEGs were associated with plant hormone biosynthesis and signal transduction (Table 3). A gene that encoded *NCED1* (Cs5g14370) and two *PP2C*s (Cs8g20420, Cs9g18020) showed an increase during fruit ripening. An *NCED2* (Cs8g14150), an *SAMD* (Cs4g02260), and two ABA 8′-hydroxylase (Cs8g05940, Cs3g23530) genes showed a decrease during fruit ripening. Some of the genes that are involved in ABA or ethylene signal transduction displayed a different expression pattern; for example, *ERF1B* (Cs5g29870) and *PYL4* (Cs7g30500) showed a decrease during fruit ripening; however, *PP2Cs* showed an increase during fruit ripening (Table 3). Some of the genes involved in sucrose and cell-wall metabolism also underwent a large change during fruit ripening, including those coding for sucrose synthase (Cs6g15930), sucrose-phosphate synthase (Cs5g19060), citrus sucrose transporter 1 (Cs3g22560), five *XET*s (Cs4g03200, Cs4g03220, Cs4g03130, Cs4g03060, Cs4g03210), seven pectinesterase genes (orange1.1t02719, Cs4g15560, Cs4g06650, Cs4g06630, Cs3g14650, Cs3g14610, Cs2g07660) and two *PG*s (orange1.1t00402, Cs9g03730), among others (Table 3). Some homologues of β-glucosidase were identified in this study; these homologues displayed different expression patterns (Tables 2 and 4). In addition, four α-1,4-galacturonosyltransferase (GAUT) genes were differentially expressed among different ripening stages in both WT and MT and exhibited decreased expression during fruit ripening (Table 3). Interestingly, 11 heat-shock proteins (HSPs) were identified, most of which were upregulated at 190 DAF in MT; however, these HSPs underwent little change in WT (Table 3). Another four l-ascorbate oxidase genes (Cs2g21220, Cs5g09230, Cs1g23090, and Cs2g29090) were identified, which were all decreased during fruit ripening in both WT and MT (Table 3).

**Table 3.** A list of some of the important differentially expressed genes between the different ripening stages in MT and WT, including genes involved in plant hormone biosynthesis/signal transduction, cell-wall metabolism, sucrose-related genes, ascorbate and aldarate metabolism, and protein processing in the endoplasmic reticulumHere, 170, 190 and 210 indicate 170, 190 and 210 DAF, respectively. The change fold is shown as a log_2_ ratio.

Gene accession	Gene description	WT fold change		MT fold change	
		190 vs 170	210 vs 190	190 vs 170	210 vs 190
Plant hormone signal transduction					
Cs8g20420	*PP2C 25*	1.25	1.37	–	2.20
Cs9g18020	*PP2C 8*	1.04	–	1.46	–
Cs4g17960	ein3-binding f-box protein 4	–1.98	–	–	–
Cs9g08850	Ethylene response 2	–1.96	1.44		1.99
Cs7g30500	*PYL4*	–1.77	–	–1.32	–
Cs4g18640	*PYL2*	–	–	11.21	–
Cs3g18000	Serine/threonine-protein kinase SRK2	–	–	1.24	–
Cs1g03300	EREBP-like factor	–	–	1.25	–
Cs8g12880	Ethylene response 3	–	–	–	1.01
Plant hormone biosynthesis					
Cs5g14370	*NCED1*	2.69	–	4.40	1.28
Cs8g05940	ABA 8′-hydroxylase	–2.44	–	–1.36	–2.15
Cs1g09250	ABA 8′-hydroxylase	–1.37		–	–
Cs8g14150	*NCED2*	–	–2.61		–2.04
Cs3g23530	ABA 8′-hydroxylase	–	–1.76	–	–
Cs7g14820	*NCED4*	–	–1.01	–	–
orange1.1t00416	*ACS*	–	–3.14	–	–2.28
Cs9g01410	*SAMD*	–	–	–	–1.11
Cs4g13870	*ACO1*	–	2.80	–	2.27
Cs4g02260	*SAMD*	–	–2.53	–1.40	–2.95
Cs2g20590	*ACO*	–	–2.58	–1.37	–
Cs2g02500	*ACO*	–	–2.41	–	–5.85
Cell-wall metabolism					
Cs4g03200	*XET*	–1.51	–	–	–1.25
Cs4g03220	*XET*	–	–3.34	3.14	–4.28
Cs4g03130	*XET*	–	–3.28	1.89	–
Cs4g03060	*XET*	–	–	–	–11.17
Cs4g03210	*XET*	–	–	–	–3.36
orange1.1t02719	Pectinesterase 3	–1.75	–1.14	–1.82	–1.41
Cs4g15560	Pectinesterase PPE8B	–	–	1.98	–
Cs4g06650	*PME3*	–2.39	–1.44	–1.88	–3.19
Cs4g06630	Thermostable pectinesterase	–1.34	–	–	–1.41
Cs3g14650	Pectinesterase	–	–10.48	–	–
Cs3g14610	Pectinesterase	–	–	1.12	–
Cs2g07660	Pectinesterase 2.2	–1.72	–	–2.31	–2.79
orange1.1t00402	Polygalacturonase	–	–11.14	–	–
Cs9g03730	Polygalacturonase	–1.28	–	–	–
Cs2g22990	*GAUT1*	–	–1.32	–	–
Cs7g16980	*GAUT1*	–1.10	–1.74	–	–1.60
Cs5g27650	*GAUT1*	–1.15	–	–	–
Cs3g24380	*GAUT8*	–1.02	–	–	–1.17
Cs9g02570	β-Glucosidase	1.92	–	2.75	–
Cs8g08680	β-Glucosidase	1.45	–1.51	–	–
Cs7g23860	β-Glucosidase	–	–1.47	–1.09	–
Cs7g01340	β-Glucosidase	–1.89	–	–	–
Sucrose-related genes					
Cs6g15930	Sucrose synthase 2	–	–10.81	–	–
Cs5g19060	Sucrose-phosphate synthase	–	–	–1.38	–
Cs3g22560	Citrus sucrose transporter 1	–	–2.61	–	–1.22
Ascorbate and aldarate metabolism					
Cs2g21220	l-Ascorbate oxidase	–	–	–	–1.44
Cs5g09230	l-Ascorbate oxidase	–1.21	–1.49	–1.63	–2.18
Cs1g23090	l-Ascorbate oxidase	–2.22	–1.72	–1.59	–2.47
Cs2g29090	l-Ascorbate oxidase	–2.84	–3.40	–3.34	–10.30
Cs5g11560	UDPglucose 6-dehydrogenase	–1.36	–	–2.05	–
Protein processing in endoplasmic reticulum					
orange1.1t03636	HSP20 family protein	–	–	2.44	–
orange1.1t03235	HSP20 family protein	–	–	2.97	–
Cs8g19540	HSP20 family protein	–	–	–	1.12
Cs8g18360	HSP18.1A	–	–	1.14	–
Cs8g18020	HSP18.1A	–	–	1.86	–
Cs7g29040	Heat-shock 70kDa protein 1/8	1.65	–	–	–
Cs6g07320	HSP20 family protein	–	–	1.45	–
Cs5g04230	HSP20 family protein	–	–11.03	–	–
Cs4g05880	HSP20 family protein	–	–	1.99	–
Cs2g24360	HSP20 family protein	–	–	1.49	–
Cs2g24040	HSP20 family protein	–	–	2.04	–

**Table 4.** Correlation between the expression ratios (MT/WT)The relative change in abundance (MT/WT) is shown as a log_2_ value from 170, 190, and 210 DAF orange fruit.

Protein ID	Proteins (log_2_ fold change, MT/WT)	Transcripts (log_2_ fold change, MT/WT)	GenBank BLAST annotation		
			E-value	Genbank ID	Annotation
170 DAF					
Cs7g04020.1	–0.590	1.066	8.00E–96	gi|255540985	40S ribosomal protein S9
Cs8g18020.1	–0.902	–1.522	4.00E–55	gi|315932718	HSP18.1A
Cs6g09150.1	–0.683	–1.421	5.00E–62	gi|255571441	Ferritin
orange1.1t03414.1	–1.388	–1.267	6.00E–42	gi|255563723	Early nodulin-like protein 1
Cs2g24040.1	–1.168	–1.733	2.00E–41	gi|357497003	18.2kDa class I heat-shock protein
Cs8g03550.1	–0.665	–1.620	2.00E–147	gi|356534254	Xyloglucan endotransglucosylase/ hydrolase protein 10-like
190 DAF					
Cs9g01630.1	0.614	1.559	0	gi|255556512	Polygalacturonase precursor
orange1.1t00340.1	0.932	1.019	1.00E–53	gi|255541538	Remorin
210 DAF					
Cs3g12080.1	–1.019	–2.379	1.00E–11	gi|2274917	Cu/Zn superoxide dismutase

### Verification of differentially expressed genes during fruit ripening

**Fig. 5.**
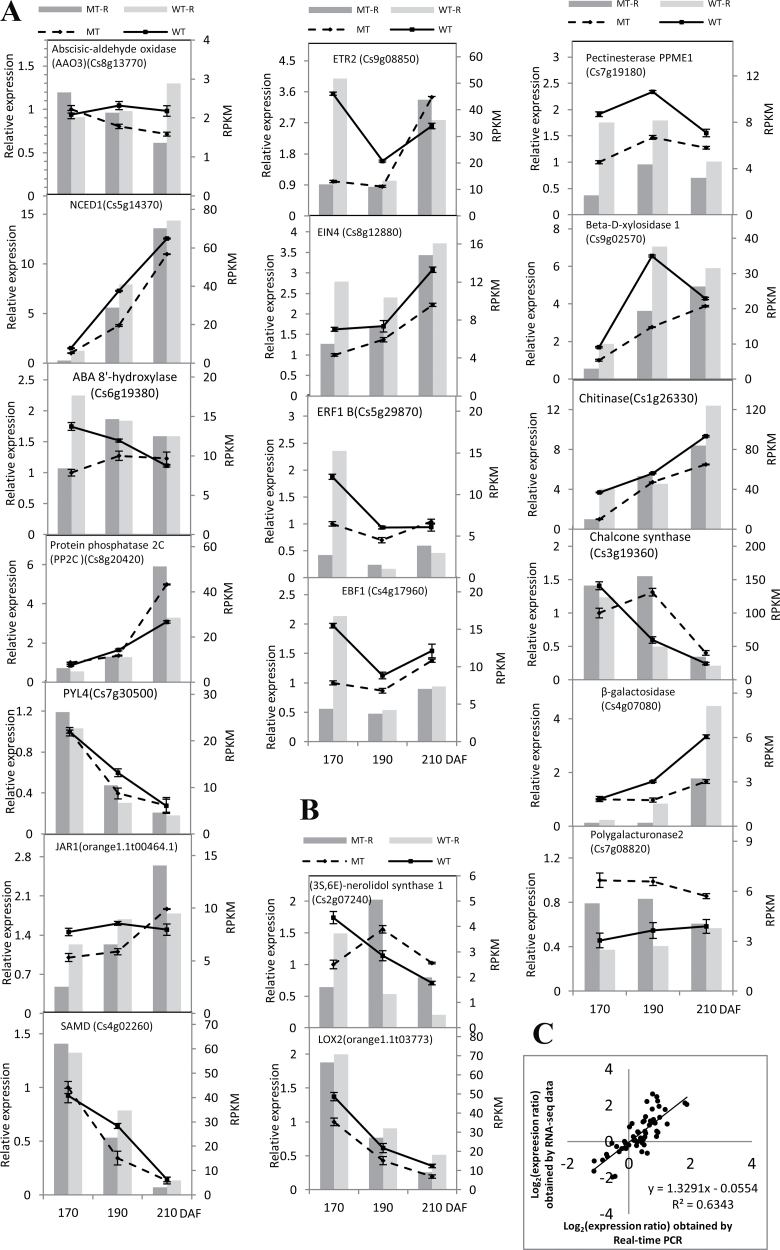
qRT-PCR validation of differential expression. (A, B) Transcript levels of 19 genes, of which 11 involve plant hormone synthesis and signal transduction (A), and eight are most likely associated with fruit development and ripening (B), in WT (solid lines) and MT (broken lines) and the corresponding expression data of RNA-seq. WT-R: the data for RNA-seq in WT; MT-R: the data for RNA-seq in MT. The *y*-axis shows the relative gene expression levels analysed by qRT-PCR. The bars represent SE (*n*=3). (C) Comparison between the gene expression ratios obtained from RNA-seq data and qRT-PCR. The RNA-seq log_2_ value of the expression ratio (*y*-axis) has been plotted against the developmental stages (*x*-axis).

Transcriptional regulation revealed by RNA-seq data was confirmed in a biologically independent experiment using a real-time quantitative reverse transcription PCR. A total of 19 genes were selected to design gene-specific primers (Supplementary Table S2 available at *JXB* online) for real-time PCR analysis (Fig. 5). A linear regression analysis showed an overall correlation coefficient of *R*=0.793, which indicates a good correlation between transcript abundance assayed by real-time PCR and the transcription profile revealed by RNA-seq data (Fig. 5C).

### Transcriptional regulation of ABA, ethylene biosynthesis, and signal transduction related genes during orange fruit development and ripening: influence of endogenous ABA levels

Through the analysis of the RNA-seq data, we found that hormone-related genes in the orange fruit ripening changed greatly, especially the genes of ABA biosynthesis and signal transduction pathways, followed by the genes of ethylene biosynthesis and signal transduction pathways. In previous studies, it has been shown that ABA and ethylene in the maturation process play important roles, and between them there is a very close interaction. To understand better the transcriptional regulation of ABA and ethylene-related genes in fruit ripening, and their relationship with the endogenous ABA levels, expression analysis of the 18 ABA-related genes and 15 ethylene-related genes was carried out together with measurement of ABA in the pulp of fruits of WT and MT.

Fruits harvested at 150, 170, 190, 210, and 240 DAF were selected. As expected, the pulp of WT fruits reached the highest ABA levels at 170 DAF, while the MT fruits reached the highest ABA levels at 190 DAF, 20 d later than WT. Although it took WT and MT a different number of days to reach the highest level of ABA, the final concentration of the highest ABA levels of these cultivars was almost equal (Fig. 1C). In the pulp of full-colour fruits from both cultivars, an important decrease in ABA content was observed, but levels in the WT fruit remained higher than that in the MT (Fig. 1A, C).

**Fig. 6.**
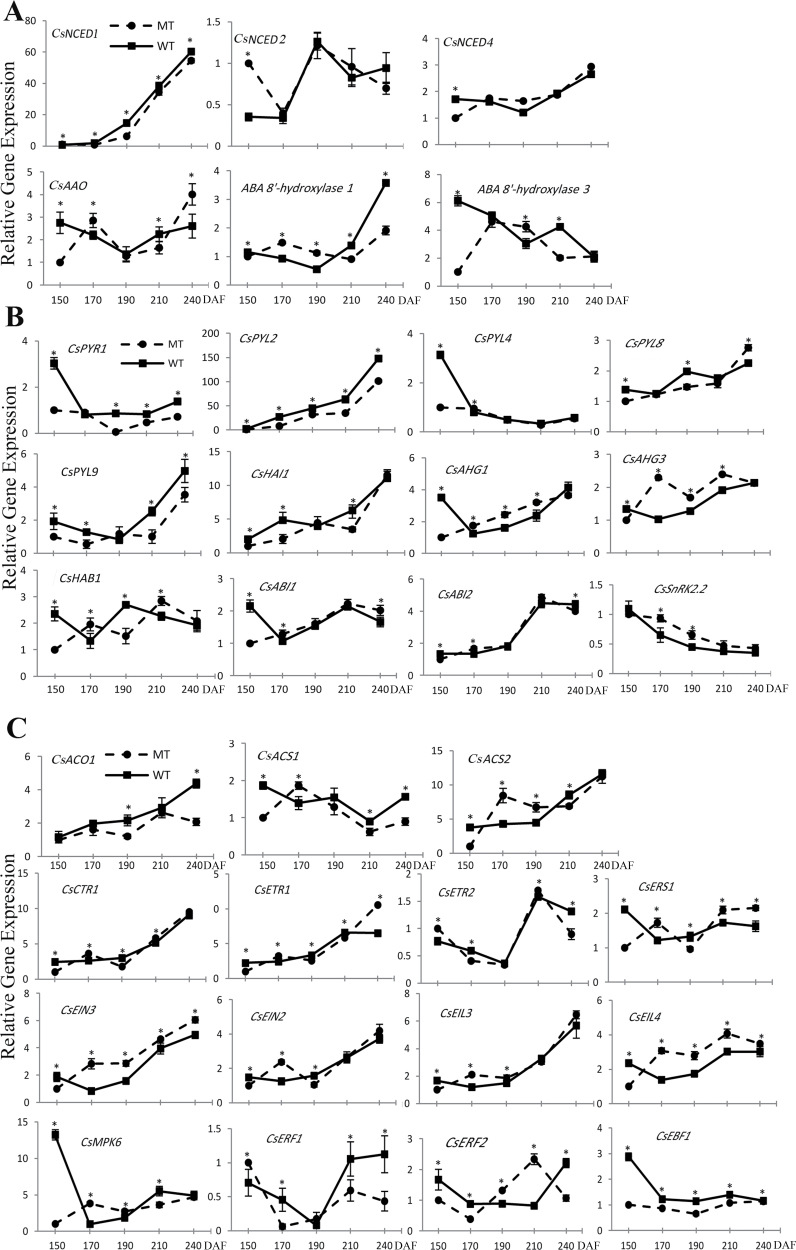
Transcript levels of genes in the ABA biosynthesis pathway (A), ABA signal transduction pathway (B,) and ethylene biosynthesis and signal transduction pathways (C). Bars represent the SE (*n*=3). An asterisk represents a statistically significant difference (*P*<0.05) analysed using Student’s *t-*test.

For the differential ABA accumulation in WT and MT during ripening, the analysis of ABA-related genes revealed a differential regulation between both cultivars, but the overall expression patterns of most of genes were the same, and the expression of some genes in MT peaked 20 d later than that in WT, such as *ABA 8′-hydroxylase 1*, *CsHAI*, and *CsHAB1* (Fig. 6A, B). NCED is a key gene in the biosynthesis of ABA, we analysed *CsNCED2* and *CsNCED4*, and found that the expression of these two genes in WT were higher than in MT at the stage of 150 DAF but had no differences at the other four stages. The expression of *CsNCED1* in WT was higher than that in MT throughout the five stages, and the expression of this gene took on a continuously upregulated trend in the two cultivars. *CsAAO*, *ABA 8′-hydroxylase 1*, and *ABA 8′-hydroxylase 3* transcript levels fluctuated during ripening in WT and MT and had significant differences between these two cultivars, especially the *ABA8′-hydroxylase 1* gene, which showed a delayed expression in MT (Fig. 6A). The expression patterns of *CsPYL2*, *CsPYL8*, *CsPYL9*, *CsABI2*, and *CsSNRK2.2* in WT and MT were the same, but their transcript levels showed differences (Fig. 6B); *CsPYR1*, *CsHAI1*, *CsAHG3*, and *CsHAB1* differed in expression patterns and expression levels between WT and MT, and the expression of these genes in MT showed a typical delay, which was very consistent with the late-ripening characteristic of MT. The expression of *CsPYL4*, *CsAHG1*, and *CsABI1* in WT were 2-fold higher than that in MT at 150 DAF stage, but in the other stages (190–240 DAF), these genes showed minor differences between both cultivars (Fig. 6B).

The analysis of the ethylene-related genes revealed a mixed expression pattern between both cultivars. Some gene expression patterns were consistent between both cultivars, such as *CsCTR1* and *CsEIL3*, while other gene expression patterns were largely different between both cultivars, such as *CsASC2* and *CsMPK6* (Fig. 6C). *ACO1*, *ASC1*, and *ASC2* are the key genes for the biosynthesis of ethylene. *CsACO1* was continuously upregulated in expression in both cultivars, and the expression levels in WT were higher than that in MT at 190 and 240 DAF (Fig. 6C). *CsASC1* and *CsASC2* in MT reached maximum expression levels at 170 DAF. At 150, 210, and 240 DAF, the transcript levels of these two genes in MT were lower than those in WT (Fig. 6C). The expression patterns of minor genes of the ethylene signal transduction pathway were similar, but the majority of genes had differences between WT and MT, such as *CsERS1*, *CsEIN3*, *CsEIN2*, *CsEIL4*, *CsERF1*, and *CsEBF1*. *CsMPK6* and *CsERF2* had differences between WT and MT, and the expression level of *CsMPK6* in WT was 13-fold higher than that in MT at 150 DAF. The transcript level of *CsERF2* in MT reached a maximum at 210 DAF stage and it was 2-fold higher than that in WT, but the expression of *CsERF2* was higher in WT at 150, 170, and 240 DAF (Fig. 6C).

### Proteomic analysis

**Fig. 7.**
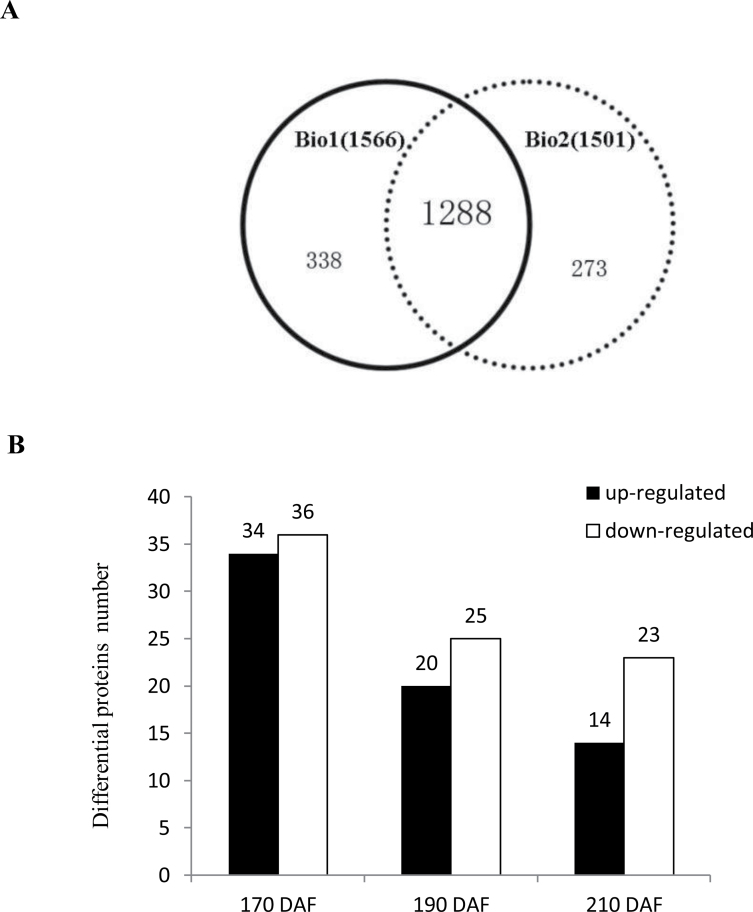
Venn diagram showing the overlap of identified proteins in the two biological repeats (A) and the number of differential proteins between MT and WT during fruit ripening (B).

In a parallel analysis, a comparative proteome survey was performed on MT and WT by the iTRAQ technique to complement the transcriptome study. A total of 1566 and 1501 proteins were identified in the two biological experiments, respectively (Supplementary Table S9 available at *JXB* online). For both of the biological repeats, the FDR was less than 0.67% (0.47% for experiment 1 and 0.67% for experiment 2). A total of 1839 proteins were identified by merging the data obtained from the two biological replicates, with an overlap of more than 78% (Fig. 7A).

**Fig. 8.**
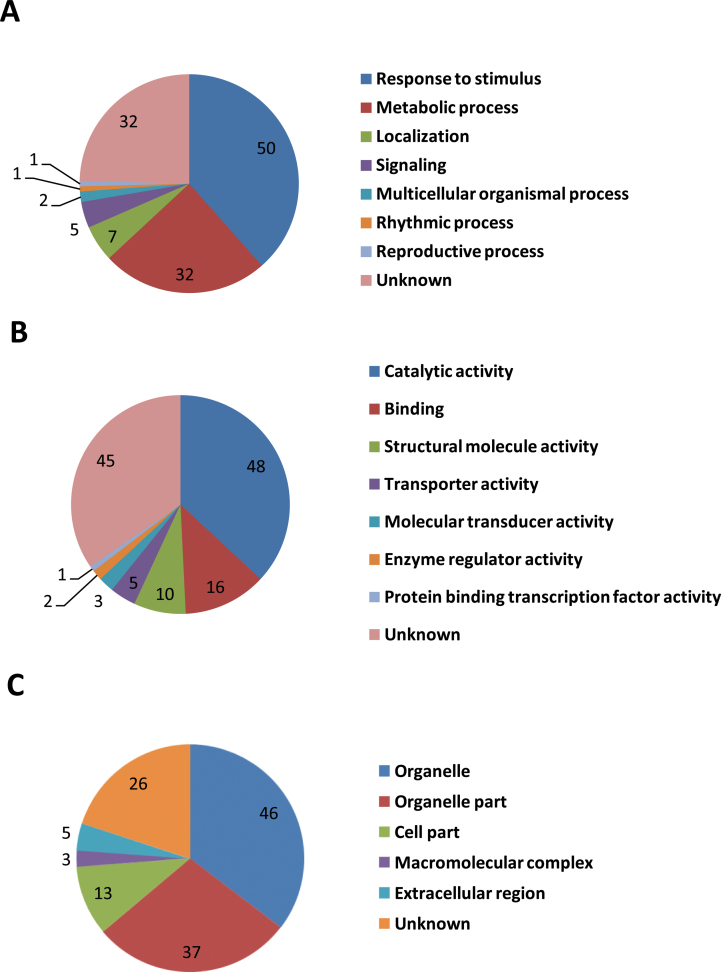
Functional categorization of the 130 differential proteins between MT and WT. The proteins were categorized based on GO annotation, and the number of each category is displayed based for biological processes (A), molecular functions (B), and cellular components (C). (This figure is available in colour at *JXB* online.)

According to previous studies (Zhu *et al.*, 2009; Lan *et al.*, 2011), regulated proteins are based on a 1.2–1.5-fold change threshold. We used a 1.5-fold cut-off to designate changes in abundance as significant for the regulated proteins. Using these criteria, a total of 130 proteins were classified as being differentially expressed between MT and WT (Supplementary Table S10 available at *JXB* online). In general, we observed that, across the three stages analysed, fruits at the 170 DAF stage showed the highest number of differentially abundant proteins in MT compared with WT. This total could be subdivided into 70, 45, and 37 proteins that vary in abundance at 170, 190 and 210 DAF, respectively (Fig. 7B). The number of downregulated proteins was greater than the number of upregulated proteins at the three ripening stages, which was consistent with the RNA-seq data (Fig. 3). To obtain functional information about the 130 differential proteins identified, the biological processes and cellular components and molecular functions based on the Blast2GO program was searched. The results of the biological process categories showed that the differential proteins were mainly distributed in response to stimulus (50), metabolic process (32), localization (7), and signalling (5), indicating that these processes play a leading role in fruit ripening (Fig. 8A). Catalytic activity (48), binding (16), structural molecule activity (10), and transporter activity (5) were the the most abundant molecular function categories, which implied that catalysis and transportation of substrates are vital for the fruit ripening (Fig. 8B). For cellular components, the organelle-related component was the largest group of proteins (83) (Fig. 8C). These results showed that the distributions of the differential proteins in functional categorization were consistent with the DEGs at the transcription level, which were also mainly distributed in categories of metabolic process, response to stimulus, organelle, and catalytic activity (Fig. 4).

As shown in Supplementary Fig. S1 available at *JXB* online, there were 14, 5, and 8 differential proteins that were involved in ‘post-translational modification, protein turnover, chaperones’ of the Clusters of Orthologous Groups (COG) proteins, at 170, 190, and 210 DAF, respectively, and there were more downregulated proteins than upregulated proteins (16 downregulated, five upregulated), including seven HSPs (Cs7g12130.1, Cs9g19220.1, Cs8g18020.1, Cs8g19490.1, Cs2g24040.1, Cs8g18360.1, Cs1g12560.1), two glutathione *S*-transferase (orange1.1t03730.1, Cs6g03820.1), one zinc finger protein (orange1.1t02815.1), one peroxiredoxin (Cs6g13880.1), two cysteine protease (Cs4g07410.1, Cs3g23180.1), five proteasome regulatory subunit (Cs1g08770.1, Cs3g14360.1, Cs1g25690.1, Cs7g07630.1, Cs8g16970.2) and three other proteins (Supplementary Table S10 available at *JXB* online). Next, there were 12, 12, 12, and 9 differential proteins between MT and WT involved in ‘general function prediction only’, ‘carbohydrate transport and metabolism’, ‘energy production and conversion’, and ‘translation, ribosomal structure and biogenesis’, respectively (Supplementary Fig. S1 and Table S10 available at *JXB* online). Notably, some proteins have been reported to be related to ripening, such as pectin methylesterase (Cs1g16560.1, Cs1g16550.1), citrate synthase (orange1.1t01588.1), and malic enzyme (Cs4g19200.1, Cs4g15270.1), which were all identified as differential proteins in MT compared with WT.

**Fig. 9.**
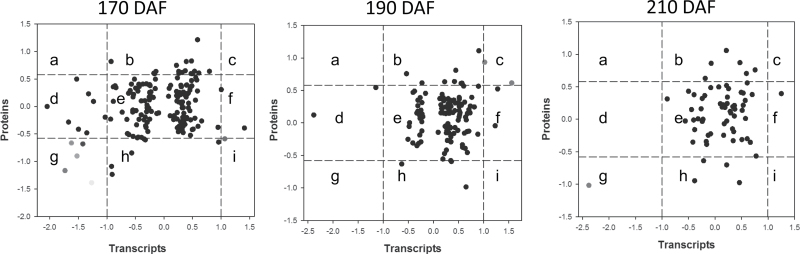
Comparison of changes in mRNA and cognate protein abundance. The relative change in abundance (MT/WT) is shown on a log_2_ scale from samples from 170, 190, and 210 DAF fruits. Each colour denotes an mRNA:protein ratio: brown, 40S ribosomal protein S9 (Cs7g04020.1); orange, HSP18.1A (Cs8g18020.1); blue, ferritin (Cs6g09150.1); yellow, early nodulin-like protein 1 (orange1.1t03414.1); red, 18.2kDa class I heat-shock protein (Cs2g24040.1); light blue, xyloglucan endotransglucosylase/hydrolase protein 10-like (Cs8g03550.1); green, remorin (orange1.1t00340.1); deep red, polygalacturonase precursor (Cs9g01630.1); and pink, Cu/Zn superoxide dismutase (Cs3g12080.1). (This figure is available in colour at *JXB* online.)

As the transcript data were obtained from exactly the same powdered fruit pulp samples, we determined the number of identified proteins for which corresponding transcripts were represented in the RNA-seq data. The distribution of the corresponding mRNA:protein ratios is shown by a scatterplot analysis of the log_2_-transformed ratios (Fig. 9). Of the 130 identified differential proteins, 54 had corresponding transcripts in the RNA-seq data. As shown in Fig. 9, almost all of the mRNA:protein ratios were concentrated at the centre of the plot (quadrant e), where protein and mRNA levels did not vary above 1.5- and 2-fold, respectively. Off centre, a total of nine mRNA:protein ratios across all three stages were found in which both the mRNA and protein levels exceeded this level of variation (Table 4, Fig. 9). The 170 DAF stage was characterized by relatively more mRNA:protein ratios falling into the quadrants a, c, g, and i compared with the 190 and 210 DAF stages, where the mRNA:protein ratios were substantially different. Moreover, five downregulated proteins, which reflected significant downregulation at the transcript level, were detected, namely, HSP18.1A (Cs8g18020.1), ferritin (Cs6g09150.1), early nodulin-like protein 1 (orange1.1t03414.1), 18.2kDa class I HSP (Cs2g24040.1), and xyloglucan endotransglucosylase/hydrolase protein 10-like (Cs8g03550.1). However, contrasting levels were observed for one gene, which exhibited upregulated expression of 40S ribosomal protein S9 (Cs7g04020.1) but downregulation of its protein abundance. At 190 DAF, we observed that mRNA:protein ratios fell mainly in quadrants b, e, and h. Therefore, only a few mRNA:protein ratios reflected significant changes at both the transcript and protein levels. Only two proteins displayed upregulation at both levels: polygalacturonase precursor (Cs9g01630.1) and remorin (orange1.1t00340.1). The 210 DAF stage was associated with the fewest mRNA:protein ratios outside of the central quadrant, and only one protein fell in the g quadrant: Cu/Zn superoxide dismutase (Cs3g12080.1).

As shown in Fig. 9, a total of 59 mRNA:protein ratios across all three stages were found to fall in quadrants b, d, h, and f, where the mRNA:protein ratios reflected significant changes at one level, the transcript or protein level, and showed a poor correlation between the transcripts and proteins. In summary, based on these results, a substantial degree of post-transcriptional regulatory activity during citrus fruit maturation is proposed, which has not been described in previous studies that focused solely on transcript or protein analysis.

## Discussion

Fruit ripening is a highly coordinated, genetically programmed, and irreversible phenomenon that involves a series of physiological, biochemical, and organoleptic changes that lead to the development of a soft, edible ripe fruit with desirable attributes (Prasanna *et al.*, 2007). In the present study, RNA-seq and iTRAQ technologies were used to investigate the differences in the transcriptome and proteome between the late-ripening bud MT and its WT. Hundreds of genes that were differently regulated during the three stages of citrus fruit ripening were identified by transcriptomic profiling. A total of 130 proteins identified by iTRAQ showed variations in abundance during ripening in MT when compared with WT. The stage 170 DAF was associated with the largest number of differently expressed proteins with respect to WT. Of the 130 identified varying proteins, 54 corresponded to gene sequences that were obtained by RNA-seq, which enabled a comparison of ripening-related differences in specific transcripts or cognate proteins. In the present study, 68.5% (37 out of 54) of the protein and transcript pairs decreased or increased in parallel during ripening. This correlation was especially apparent at the early ripening stage (170 DAF). A large number of genes showed consistency between the transcript and protein levels, whereas those genes that exhibited inconsistency between these levels suggest that post-transcriptional regulation plays an important role in the regulation of fruit ripening. Both the transcriptomic and proteomic data are important in deciphering the molecular processes involved in fruit ripening. The integrative transcriptomic and proteomic data not only highlighted a set of genes/proteins that were possibly involved in the late-ripening trait formation of MT but also revealed molecular characterizations associated with fruit development and ripening in sweet oranges.

### Identification of potential regulators and metabolism pathways involved in fruit ripening

**Fig. 10.**
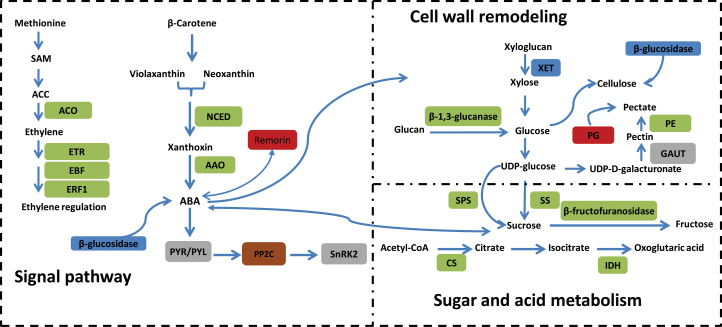
Summary of some of the biological pathways involved in citrus fruit ripening. Red boxes indicate genes/proteins that were upregulated in MT compared with WT, brown boxes indicate genes/proteins that were upregulated during fruit ripening in WT, green boxes indicate genes/proteins that were downregulated in MT compared with WT, grey boxes indicate genes/proteins that were downregulated during fruit ripening in WT, and blue boxes represent genes/proteins that were shown to have mixed expression patterns (both up- and downregulation) in MT compared with WT. *ACO*, 1-aminocyclopropane-1-carboxylate oxidase*; ETR*, ethylene receptor; *EBF*, EIN3-binding F-box protein; *ERF1*, ethylene-responsive transcription factor; *NCED*, 9-cis-epoxycarotenoid dioxygenase; *AAO*, abscisic-aldehyde oxidase; *PYR/PYL*, abscisic acid receptor; *PP2C*, protein phosphatase 2C; *SnRK2*, serine/threonine-protein kinase SRK2; *XET*, xyloglucan endotransglycosylase; *PG*, polygalacturonase; *PE*, pectinesterase; *GAUT*, alpha-1,4-galacturonosyltransferase; *SPS*, sucrose-phosphate synthase; *SS*, sucrose synthase; *CS*, citrate synthase; and *IDH*, isocitrate dehydrogenase. (This figure is available in colour at *JXB* online.)

Some of the regulated genes and proteins in the most important pathways are shown in Fig. 10. Among these, the ABA pathway may play a central role in orange fruit development and ripening, and this regulation may function in combination with other hormones, including ethylene. We also observed upstream and/or downstream biological processes, such as sugar metabolism and cell-wall biosynthesis, that were significantly changed during fruit ripening. These data depict a detailed picture of the regulation network involved in orange fruit ripening.

The phytohormone ABA is a major endogenous factor and is the primary signal that regulates fruit development, maturation, and senescence (Gosti *et al.*, 1999; Romero *et al.*, 2012; Yun *et al.*, 2012; Jia *et al.*, 2013b). In non-climacteric fruit, ripening is thought to be regulated by ABA, with ethylene also playing role (Koyama *et al.*, 2010; Ji *et al.*, 2012; Paul *et al.*, 2012). The ABA level in plants is regulated by the pathways of ABA biosynthesis and degradation. *NCED*s and *AAO*s are the key rate-limiting steps in ABA biosynthesis; ABA 8′-hydroxylase is a key gene for the catabolism of ABA (Seo *et al.*, 2000; Rodrigo *et al.*, 2006; Zhang *et al.*, 2009). In this study, *NCED*s, *AAO*s and ABA 8′-hydroxylase genes were found to be differentially expressed between MT and WT (Figs 6 and 9, Table 2). In addition, these genes were also identified to be differentially expressed in different ripening stages during fruit ripening in MT and/or WT (Figs 6 and 9, Table 3). Furthermore, the curve of ABA of MT was different from that of WT (Fig. 1C). This, we hypothesized that ABA is a key factor for the formation of late ripening in MT, and that the genes involved in ABA biosynthesis and signal transduction pathways might be the mutation source. Two full-length cDNAs (*CsNCED1* and *CsNCED2*) encoding *NCED*s were isolated and characterized from the epicarp of the orange fruits. *CsNCED1* is likely to play a primary role in the biosynthesis of ABA, and *CsNCED2* appears to play a subsidiary role that is restricted to chromoplast-containing tissue (Rodrigo *et al.*, 2006). By suppressing the expression of *SlNCED1* using an RNAi method in tomato, ABA accumulation and *SlNCED1* transcript levels were downregulated to between 20 and 50% of the levels measured in the control fruit, which led to downregulation in transcription of the genes that encode major cell-wall catabolic enzymes, such as *PG*, *PME*, *XET*, and others (Sun *et al.*, 2012). Accordingly, in our study, the *NCED1* gene was also identified as having differential expression during fruit ripening between MT and WT (Fig. 6). Thus, *NCED1* might be a significant regulator for orange fruit ripening. The initial response to ABA implies the ABA-dependent PYR/PYL-mediated inactivation of *PP2C*s, which allows for the release of *SnRK2*s and, hence, the phosphorylation of ABA-dependent transcription factors or other proteins. This ABA signal is later attenuated by upregulation of the *PP2C*s and the downregulation of the PYR/PYL genes in an ABA-dependent manner. Thus, resetting of the ABA transduction pathway offers a dynamic mechanism for modulating the ABA response (Nishimura *et al.*, 2010). Interestingly, in the present study, upregulation of the *PP2C*s genes (such as *CsABI1*, *CsABI2*, and *CsAHG1*, among others) was concomitant with downregulation of the *PYL4* gene in both WT and MT (Figs 5 and 6, Table 3). Therefore, these results suggest that a transcriptional negative-feedback regulatory mechanism might modulate the ABA responses during citrus fruit ripening, which is consistent with a previous study (Romero *et al.*, 2012). At the 190 DAF stage, we found that remorin protein was upregulation in MT at transcript and protein levels (Table 4, Fig. 10). In a previous study (Lin *et al.*, 2003), remorin homologues could be upregulated by exogenous ABA, which revealed that remorin protein was involved in the ABA signal transduction pathway. In addition, experiments *in vivo* and *in vitro* have proved that oligonucleotide galacturonic acid can significantly improve the phosphorylation level of remorin protein, and oligonucleotide galacturonic acid could influence the triggering of some physiological processes that depend on hormones (Bellincampi *et al.*, 1996).

Ethylene is also involved in the ripening of orange fruits, which are non-climacteric, and has long been known to play a major role in the ripening process of climacteric fruits (Theologis, 1992; Alexander and Grierson, 2002; Klee, 2002). Increasing evidence has indicated that ethylene is also involved in regulation of the ripening of non-climacteric fruits; even the small amount of ethylene that is produced by a fruit might be sufficient to trigger ripening-related physiological responses (Perkins-Veazie *et al.*, 1996; Chervin *et al.*, 2004; Trainotti *et al.*, 2005; Iannetta *et al.*, 2006). Experiments with 1-methylcyclopropene (an action inhibitor of ethylene) have indicated that ethylene is required for the onset of accumulation of anthocyanins, fruit swelling, and the decrease in acidity that is associated with the ripening of grape berries (Chervin *et al.*, 2004). In the present study, two genes were identified as *ACO*; these were downregulated in MT (Fig. 10, Table 2). *ACO* is a key enzyme for ethylene biosynthesis. Two *ETR*, one *EBF4*, and one *ERF1B* genes were identified as being involved in the ethylene signal transduction pathway; these genes were also downregulated in MT (Fig. 10, Table 2). Ethylene is perceived by the receptors *ETR1* and related proteins (Chang *et al.*, 1993; Hua and Meyerowitz, 1998). The ethylene signal is transduced to *EIN3* through *CTR1* (Kieber *et al.*, 1993) and *EIN2* (Alonso *et al.*, 1999). Studies have also revealed that, in the absence of ethylene, the *EIN3* protein is quickly degraded through a ubiquitin–proteasome pathway that is mediated by two F-box proteins (*EBF1* and *EBF2*), whereas the *EIN3* protein is stabilized by ethylene itself (Guo and Ecker, 2003; Potuschak *et al.*, 2003; Gagne *et al.*, 2004). Citrus (and other non-climacteric fruits) lack a ripening-associated autocatalytic rise in ethylene production. However, Goldschmidt (1997) pointed out the potential significance of even the lowest levels of ethylene present in non-climacteric fruits in regulation of the ripening process. Citrus reveal ripening-related symptoms in response to exogenous ethylene, and induction of the ripening of citrus fruit peel by ethylene was also found to be opposed by gibberellins and cytokinins, possibly due to the reduction in the tissue’s sensitivity to ethylene (Goldschmidt, 1997). Recent work on apple by Johnston *et al.* (2009) has revealed that early- and late-ripening events differ not only in their dependence on ethylene but also in their sensitivity to ethylene. In the present study, we observed that some genes that are related to ethylene biosynthesis and signal transduction were differentially expressed between MT and WT, and were also differentially expressed in the different fruit-ripening stages of WT and MT (Fig. 10, Tables 2 and 3). These findings thereby necessitate further work not only on the role of the development/developmental signals but also on the mechanism of alteration in the sensitivity of the tissue/s with the development of fruit.

Sugars, as crucial components of fruit quality, are also involved in signal transduction. The effect of sugar on many developmental processes is concentration dependent (Gibson, 2005). In the present study, the sugar content of MT was lower than that of WT (Fig. 2D–F); however, the citric acid content in MT was higher than that in WT (Fig. 2B). Furthermore, some genes/proteins that are involved in sucrose, citrate, and fructose metabolism were downregulated in MT (Fig. 10, Table 2). Interestingly, in the present study, we found that changes in the sucrose content were closely correlated with phase transitions in the developmental process that spans the mature green phase to full ripening; the content of sucrose remained low and relatively stable in the early stages of the mature green phase and increased sharply when the fruits began to enter the break phase (Fig. 2F). In the strawberry, sugars, especially sucrose, can promote the ripening of fruit (Jia *et al.*, 2011). In the present study, a citrus sucrose transporter 1 gene was identified in MT and WT; this gene was significantly changed during fruit ripening (Table 3). The RNAi of the sucrose transporter 1 (SUT1) in the strawberry was able to significantly reduce the sucrose content and delayed fruit ripening (Jia *et al.*, 2013a); therefore, sucrose deserves further investigation as a potentially significant regulator for citrus fruit ripening.

### Cell-wall metabolism and other gene/protein changes during fruit ripening

Cell-wall metabolism is one of the major fruit ripening-related changes; this type of metabolism involves the dismantling of multiple polysaccharide networks by diverse families of cell-wall-modifying proteins, including enzymes for pectin and cellulose catabolism. The degradation of pectin and cellulose depends on ethylene during the softening of climacteric fruits (Ergun *et al.*, 2005; Nishiyama *et al.*, 2007). Recent work has revealed that ABA affects cell-wall catabolism during fruit ripening via downregulation of the expression of major catabolic genes (*PG*, *PE*, and *XET*) (Sun *et al.*, 2012). A subset of cell-wall-modifying genes and proteins was found to be downregulated in MT and WT during fruit ripening, including *PE*s, *PG*s, and *GAUT*s (Fig. 10, Table 3). *GAUT1* is the core of synthesis of homogalacturonan, the most abundant pectic polysaccharide and the core of the plant cell-wall pectin biosynthetic homogalacturonan:galacturonosyltransferase complex (Atmodjo *et al.*, 2011). Comparing MT with WT, the genes/proteins that were identified as *PG*s were upregulated in MT and β-glucosidase genes, and *XET*s displayed mixed expression patterns (Fig. 10, Table 2). Changes in the activity of these cell-wall-related genes are known to result in the abnormal development of juice sac granulation (Sharma and Saxena, 2004), and modifications in the cell-wall structure or in the components of the membranes of the segments and juice sacs during fruit development and ripening clearly influence the formation of the fruit pulp melting characteristic (Waldron *et al.*, 2003).

A striking feature of the proteomic data was that seven proteins (Supplementary Table S10 available at *JXB* online) were identified as HSPs, and 11 differential expression HSP transcripts were also identified in MT during fruit ripening (Table 3). The possible important roles of HSPs in fruit development and ripening have been reported recently in tomato (Neta-Sharir *et al.*, 2005; Faurobert *et al.*, 2007), apple (Wang *et al.*, 2009), apricot (Grimplet *et al.*, 2005), and citrus (Pan *et al.*, 2012). In the present study, seven HSPs, including two HSP90s exhibiting increased the abundance in MT and five small HSPs exhibiting decreased the abundance in MT were identified (Supplementary Table S9 available at *JXB* online). HSPs, which function as chaperone molecules, are important in post-translational modification, and the seven HSPs identified in this study indicate possible roles in fruit ripening.

In the present study, in addition to ABA and ethylene, sucrose appeared to function as another key signal in the regulation of citrus fruit ripening. Fruit ripening is the result of an orderly alteration of a series of physiological and biochemical events, such as sugar and acid metabolism, cell-wall metabolism, and pigment and flavour metabolism, and each of these metabolic systems or processes is involved in the regulation of a number of genes. It is not known whether each metabolic system is independently regulated by a unique signalling pathway or by a common signalling network. It is unlikely that many metabolic systems across a number of developmental stages would be controlled by a single signal. Thus, it is not surprising that the development and ripening of citrus fruit may be controlled by multiple signals. However, if one signal pathway changes, then this change could affect the entire process of fruit development and ripening. Previous studies on strawberry showed that exogenous ABA promoted strawberry ripening while repressing ABA levels by the administration of an ABA biosynthesis inhibitor, which resulted in a delay in ripening and a decrease in the sugar content; in addition, treatment with glucose and especially sucrose increased the levels of ABA and promoted fruit ripening (Jia *et al.*, 2011, 2013a). These data suggest that the two signalling pathways interact cooperatively to regulate fruit ripening. In the present study, the ABA pathway, ethylene pathway, and sucrose pathway were all different between MT and WT, and we have not proven which is the most important factor for forming the late-ripening trait of MT. This issue will require further study.

## Conclusions

A multiple-level analysis of the gene/protein expression changes in MT, compared with WT, was conducted in our study. Combined with transcriptomic and proteomic data, the results revealed several key candidate regulators that may play high-priority roles in citrus fruit ripening. Based on an integrated analysis of the transcriptome and proteome, we identified biological processes that appear to be of high importance in the ripening process, such as the signal transduction of hormone, the regulation of hormone and sugar levels, and cell-wall metabolism. Thus, the integrated analysis enabled us to generate additional information for a comprehensive understanding of biological events that are relevant to the regulation of citrus fruit ripening. These results have revealed multiple ripening-associated events during citrus ripening, providing new insights into the molecular mechanism of citrus ripening regulatory networks.

## Supplementary data

Supplementary data are available at *JXB* online.

Supplementary Fig. S1. The distribution of differential proteins in the COG function categories.

Supplementary Table S1. Genes and primers for real-time PCR.

Supplementary Table S2. Primer sequences for real-time PCR.

Supplementary Table S3. An integration of differentially expressed genes between MT and WT.

Supplementary Table S4. Genes that were differentially expressed between MT and WT at each of the three fruit developmental stages.

Supplementary Table S5. The KEGG pathways of more than three differentially expressed genes between MT and WT at 170, 190, and 210 DAF.

Supplementary Table S6. Several important KEGG pathways involved in MT and WT.

Supplementary Table S7. Differentially expressed genes of MT at three development stages.

Supplementary Table S8. Differentially expressed genes of WT at three development stages.

Supplementary Table S9. The total identified proteins of two biological repeats.

Supplementary Table S10. Differential proteins between WT and MT at three development stages.

Supplementary Results.

Supplementary Data

## Abbreviations:

ABA: abscisic acid
COG: Clusters of Orthologous Groups
DAF: days after flowering
DEG: differentially expressed genes
FDR: false discovery rate
GO: Gene Ontology
HCD: high-energy collision dissociation
HPLC-ESI-MS/MS: high-performance liquid chromatography electrospray ionization tandem mass spectrometry
HSP: heat-shock protein
iTRAQ: isobaric tags for relative and absolute quantitation
KEGG: Kyoto Encyclopaedia of Genes and Genomes
MT: mutant
RNAi: RNA interference
RNA-seq: RNA sequencing
SCX: strong cationic exchange
TOA: total organic acid
TSS: total soluble sugar
WT: wild type.

## References

[CIT0001] Alexander L Grierson D 2002 Ethylene biosynthesis and action in tomato: a model for climacteric fruit ripening. Journal of Experimental Botany 53, 2039–2055 12324528 10.1093/jxb/erf072

[CIT0002] Alonso JM Hirayama T Roman G Nourizadeh S Ecker JR 1999 EIN2, a bifunctional transducer of ethylene and stress responses in Arabidopsis. Science 284, 2148–2152 10381874 10.1126/science.284.5423.2148

[CIT0003] Atmodjo MA Sakuragi Y Zhu X Burrell AJ Mohanty SS Atwood JA III Orlando R Scheller HV Mohnen D 2011 Galacturonosyltransferase (GAUT)1 and GAUT7 are the core of a plant cell wall pectin biosynthetic homogalacturonan: galacturonosyltransferase complex. Proceedings of the National Academy of Sciences, USA 108, 20225–20230 10.1073/pnas.1112816108 PMC3250160 22135470

[CIT0004] Bain J 1958 Morphological, anatomical, and physiological changes in the developing fruit of the Valencia orange, *Citrus sinensis* (L) Osbeck. Australian Journal of Botany 6, 1–23

[CIT0005] Bellincampi D Cardarelli M Zaghi D Serino G Salvi G Gatz C Cervone F Altamura MM Costantino P Lorenzo GD 1996 Oligogalacturonides prevent rhizogenesis in rolB-transformed tobacco explants by inhibiting auxin-induced expression of the rolB gene. Plant Cell 8, 477–487 12239391 10.1105/tpc.8.3.477 PMC161114

[CIT0006] Chang C Kwok SF Bleecker AB Meyerowitz EM 1993 Arabidopsis ethylene-response gene ETR1: similarity of product to two-component regulators. Science 262, 539–544 8211181 10.1126/science.8211181

[CIT0007] Chervin C El-Kereamy A Roustan JP Latche A Lamon J Bouzayen M 2004 Ethylene seems required for the berry development and ripening in grape, a non-climacteric fruit. Plant Science 167, 1301–1305

[CIT0008] Elias JE Gygi SP 2007 Target-decoy search strategy for increased confidence in large-scale protein identifications by mass spectrometry. Nature Methods 4, 207–214 17327847 10.1038/nmeth1019

[CIT0009] Ergun M Jeong JW Huber DJ Cantliffe DJ 2005 Suppression of ripening and softening of ‘Galia’ melons by 1-methylcyclopropene applied at preripe or ripe stages of development. Hortscience 40, 170–175

[CIT0010] Fan X Mattheis JP Fellman JK 1998 A role for jasmonates in climacteric fruit ripening. Planta 204, 444–449

[CIT0011] Faurobert M Mihr C Bertin N Pawlowski T Negroni L Sommerer N Causse M 2007 Major proteome variations associated with cherry tomato pericarp development and ripening. Plant Physiology 143, 1327–1346 17208958 10.1104/pp.106.092817 PMC1820912

[CIT0012] Gagne JM Smalle J Gingerich DJ Walker JM Yoo SD Yanagisawa S Vierstra RD 2004 Arabidopsis EIN3-binding F-box 1 and 2 form ubiquitin-protein ligases that repress ethylene action and promote growth by directing EIN3 degradation. Proceedings of the National Academy of Sciences, USA 101, 6803–6808 10.1073/pnas.0401698101 PMC404126 15090654

[CIT0013] Gan CS Chong PK Pham TK Wright PC 2007 Technical, experimental, and biological variations in isobaric tags for relative and absolute quantitation (iTRAQ). Journal of Proteome Research 6, 821–827 17269738 10.1021/pr060474i

[CIT0014] Gibson SI 2005 Control of plant development and gene expression by sugar signaling. Current Opinion in Plant Biology 8, 93–102 15653406 10.1016/j.pbi.2004.11.003

[CIT0015] Goldschmidt EE 1997 Ripening of citrus and other non-climacteric fruits: a role for ethylene. In: *VIII International Symposium on Plant Bioregulation in Fruit Production* . ISHS Acta Horticulturae 463, 335–340

[CIT0016] Gosti F Beaudoin N Serizet C Webb AA Vartanian N Giraudat J 1999 ABI1 protein phosphatase 2C is a negative regulator of abscisic acid signaling. Plant Cell 11, 1897–1910 10521520 10.1105/tpc.11.10.1897 PMC144098

[CIT0017] Grimplet J Romieu C Audergon JM Marty I Albagnac G Lambert P Bouchet JP Terrier N 2005 Transcriptomic study of apricot fruit (*Prunus armeniaca*) ripening among 13 006 expressed sequence tags. Physiologia Plantarum 125, 281–292

[CIT0018] Guo H Ecker JR 2003 Plant responses to ethylene gas are mediated by SCF(EBF1/EBF2)-dependent proteolysis of EIN3 transcription factor. Cell 115, 667–677 14675532 10.1016/s0092-8674(03)00969-3

[CIT0019] Hua J Meyerowitz EM 1998 Ethylene responses are negatively regulated by a receptor gene family in *Arabidopsis thaliana* . Cell 94, 261–271 9695954 10.1016/s0092-8674(00)81425-7

[CIT0020] Iannetta PPM Laarhoven LJ Medina-Escobar N James EK McManus MT Davies HV Harren FJM 2006 Ethylene and carbon dioxide production by developing strawberries show a correlative pattern that is indicative of ripening climacteric fruit. Physiologia Plantarum 127, 247–259

[CIT0021] Ji K Chen P Sun L 2012 Non-climacteric ripening in strawberry fruit is linked to ABA, FaNCED2 and FaCYP707A1. Functional Plant Biology 39, 351–357 10.1071/FP11293 32480787

[CIT0022] Jia H Wang Y Sun M 2013 *a* Sucrose functions as a signal involved in the regulation of strawberry fruit development and ripening. New Phytologist 198, 453–465 23425297 10.1111/nph.12176

[CIT0023] Jia HF Chai YM Li CL Lu D Luo JJ Qin L Shen YY 2011 Abscisic acid plays an important role in the regulation of strawberry fruit ripening. Plant Physiology 157, 188–199 21734113 10.1104/pp.111.177311 PMC3165869

[CIT0024] Jia HF Lu D Sun JH Li CL Xing Y Qin L Shen YY 2013 *b* Type 2C protein phosphatase ABI1 is a negative regulator of strawberry fruit ripening. Journal of Experimental Botany 64, 1677–1687 23404898 10.1093/jxb/ert028 PMC3617833

[CIT0025] Johnston JW Gunaseelan K Pidakala P Wang M Schaffer RJ 2009 Co-ordination of early and late ripening events in apples is regulated through differential sensitivities to ethylene. Journal of Experimental Botany 60, 2689–2699 19429839 10.1093/jxb/erp122 PMC2692014

[CIT0026] Katz E Boo KH Kim HY Eigenheer RA Phinney BS Shulaev V Negre-Zakharov F Sadka A Blumwald E 2011 Label-free shotgun proteomics and metabolite analysis reveal a significant metabolic shift during citrus fruit development. Journal of Experimental Botany 62, 5367–5384 21841177 10.1093/jxb/err197 PMC3223037

[CIT0027] Kieber JJ Rothenberg M Roman G Feldmann KA Ecker JR 1993 CTR1, a negative regulator of the ethylene response pathway in Arabidopsis, encodes a member of the raf family of protein kinases. Cell 72, 427–441 8431946 10.1016/0092-8674(93)90119-b

[CIT0028] Klee HJ 2002 Control of ethylene-mediated processes in tomato at the level of receptors. Journal of Experimental Botany 53, 2057–2063 12324529 10.1093/jxb/erf062

[CIT0029] Koyama K Sadamatsu K Goto-Yamamoto N 2010 Abscisic acid stimulated ripening and gene expression in berry skins of the Cabernet Sauvignon grape. Functional & Integrative Genomics 10, 367–381 19841954 10.1007/s10142-009-0145-8

[CIT0030] Lan P Li W Wen TN Shiau JY Wu YC Lin W Schmidt W 2011 iTRAQ protein profile analysis of Arabidopsis roots reveals new aspects critical for iron homeostasis. Plant Physiology 155, 821–834 21173025 10.1104/pp.110.169508 PMC3032469

[CIT0031] Li HS 2000 Principles and techniques of plant physiology and biochemistry experiment. Beijing: Higher Education Press, 134–137

[CIT0032] Lin F Xu SL Ni WM Chu ZQ Xu ZH Xue HW 2003 Identification of ABA-responsive genes in rice shoots via cDNA macroar. Cell Res 13, 59–68 12643350 10.1038/sj.cr.7290151

[CIT0033] Liu Q Zhu A Chai LJ Zhou WJ Yu KQ Ding J Xu J Deng XX 2009 Transcriptome analysis of a spontaneous mutant in sweet orange [*Citrus sinensis* (L.) Osbeck] during fruit development. Journal of Experimental Botany 60, 801–813 19218315 10.1093/jxb/ern329 PMC2652045

[CIT0034] Liu Y Liu Q Tao N 2006 *a* Efficient isolation of RNA from fruit peel and pulp of ripening navel orange (*Citrus sinensis* Osbeck). Journal of Huazhong Agricultural University 25, 300–304

[CIT0035] Liu YZ Tang P Tao NG Xu Q Peng SA Deng XX Xiang KS Huang RH 2006 *b* Fruit coloration difference between Fengwan, a late-maturing mutant and its original cultivar Fengjie72-1 of navel orange *(Citrus sinensis* Osbeck). Journal of Plant Physiology and Molecular Biology 32, 31–36 16477128

[CIT0036] Mortazavi A Williams BA McCue K Schaeffer L Wold B 2008 Mapping and quantifying mammalian transcriptomes by RNA-Seq. Nature Methods 5, 621–628 18516045 10.1038/nmeth.1226 PMC13303166

[CIT0037] Neta-Sharir I Isaacson T Lurie S Weiss D 2005 Dual role for tomato heat shock protein 21: Protecting photosystem II from oxidative stress and promoting color changes during fruit maturation. Plant Cell 17, 1829–1838 15879560 10.1105/tpc.105.031914 PMC1143080

[CIT0038] Nishimura N Sarkeshik A Nito K 2010 PYR/PYL/RCAR family members are major in-vivo ABI1 protein phosphatase 2C-interacting proteins in Arabidopsis. Plant Journal 61, 290–299 19874541 10.1111/j.1365-313X.2009.04054.x PMC2807913

[CIT0039] Nishiyama K Guis M Rose JKC 2007 Ethylene regulation of fruit softening and cell wall disassembly in Charentais melon. Journal of Experimental Botany 58, 1281–1290 17308329 10.1093/jxb/erl283

[CIT0040] Noirel J Evans C Salim M Mukherjee J Ow SY Pandhal J Pham TK Biggs CA Wright PC 2011 Methods in quantitative proteomics: setting iTRAQ on the right track. Current Proteomics 8, 17–30

[CIT0041] Osorio S Alba R Damasceno CM 2011 Systems biology of tomato fruit development: combined transcript, protein, and metabolite analysis of tomato transcription factor (nor, rin) and ethylene receptor (Nr) mutants reveals novel regulatory interactions. Plant Physiology 157, 405–425 21795583 10.1104/pp.111.175463 PMC3165888

[CIT0042] Pan X Welti R Wang X 2010 Quantitative analysis of major plant hormones in crude plant extracts by high-performance liquid chromatography-mass spectrometry. Nature Protocols 5, 986–992 10.1038/nprot.2010.37 20448544

[CIT0043] Pan Z Liu Q Yun Z Guan R Zeng W Xu Q Deng X 2009 Comparative proteomics of a lycopene-accumulating mutant reveals the important role of oxidative stress on carotenogenesis in sweet orange (*Citrus sinensis* [L.] osbeck). Proteomics 9, 5455–5470 19834898 10.1002/pmic.200900092

[CIT0044] Pan ZY Zeng YL An JY Ye JL Xu Q Deng XX 2012 An integrative analysis of transcriptome and proteome provides new insights into carotenoid biosynthesis and regulation in sweet orange fruits. Journal of Proteomics 75, 4879–4880 10.1016/j.jprot.2012.03.016 22472342

[CIT0045] Paul V Pandey R Srivastava GC 2012 The fading distinctions between classical patterns of ripening in climacteric and non-climacteric fruit and the ubiquity of ethylene—an overview. Journal of Food Science and Technology-Mysore 49, 1–21 10.1007/s13197-011-0293-4 PMC3550874 23572821

[CIT0046] Perkins-Veazie PM Huber DJ Brecht JK 1996 In vitro growth and ripening of strawberry fruit in the presence of ACC, STS or propylene. Annals of Applied Biology 128, 105–116

[CIT0047] Potuschak T Lechner E Parmentier Y Yanagisawa S Grava S Koncz C Genschik P 2003 EIN3-dependent regulation of plant ethylene hormone signaling by two Arabidopsis F box proteins: EBF1 and EBF2. Cell 115, 679–689 14675533 10.1016/s0092-8674(03)00968-1

[CIT0048] Prasanna V Prabha TN Tharanathan RN 2007 Fruit ripening phenomena—an overview. Critical Reviews in Food Science and Nutrition 47, 1–19 17364693 10.1080/10408390600976841

[CIT0049] Rodrigo MJ Alquezar B Zacarias L 2006 Cloning and characterization of two 9-*cis*-epoxycarotenoid dioxygenase genes, differentially regulated during fruit maturation and under stress conditions, from orange (*Citrus sinensis* L. Osbeck). Journal of Experimental Botany 57, 633–643 16396998 10.1093/jxb/erj048

[CIT0050] Rodrigo MJ Marcos JF Alférez F Mallent MD Zacarías L 2003 Characterization of Pinalate, a novel *Citrus sinensis* mutant with a fruit-specific alteration that results in yellow pigmentation and decreased ABA content. Journal of Experimental Botany 54, 727–738 12554716 10.1093/jxb/erg083

[CIT0051] Rolland F Baena-Gonzalez E Sheen J 2006 Sugar sensing and signaling in plants: conserved and novel mechanisms. Annual Review of Plant Biology 57, 675–709 10.1146/annurev.arplant.57.032905.105441 16669778

[CIT0052] Romero P Lafuente MT Rodrigo MJ 2012 The Citrus ABA signalosome: identification and transcriptional regulation during sweet orange fruit ripening and leaf dehydration. Journal of Experimental Botany 63, 4931–4945 22888124 10.1093/jxb/ers168 PMC3428003

[CIT0053] Seo M Koiwai H Akaba S Komano T Oritani T Kamiya Y Koshiba T 2000 Abscisic aldehyde oxidase in leaves of *Arabidopsis thaliana* . The Plant Journal 23, 481–488 10972874 10.1046/j.1365-313x.2000.00812.x

[CIT0054] Sharma RR Saxena SK 2004 Rootstocks influence granulation in Kinnow mandarin (*Citrus nobilis*×*C. deliciosa* ). Scientia Horticulturae 101, 235–242

[CIT0055] Smeekens S 2000 Sugar-induced signal transduction in plants. Annual Review of Plant Physiology and Plant Molecular Biology 51, 49–81 10.1146/annurev.arplant.51.1.49 15012186

[CIT0056] Soto A Ruiz KB Ravaglia D Costa G Torrigiani P 2013 ABA may promote or delay peach fruit ripening through modulation of ripening- and hormone-related gene expression depending on the developmental stage. Plant Physiology and Biochemistry 64, 11–24 23337357 10.1016/j.plaphy.2012.12.011

[CIT0057] Sun L Sun YF Zhang M 2012 Suppression of 9-*cis*-epoxycarotenoid dioxygenase, which encodes a key enzyme in abscisic acid biosynthesis, alters fruit texture in transgenic tomato. Plant Physiology 158, 283–298 22108525 10.1104/pp.111.186866 PMC3252109

[CIT0058] Theologis A 1992 One rotten apple spoils the whole bushel: the role of ethylene in fruit ripening. Cell 70, 181–184 1638627 10.1016/0092-8674(92)90093-r

[CIT0059] Trainotti L Pavanello A Casadoro G 2005 Different ethylene receptors show an increased expression during the ripening of strawberries: does such an increment imply a role for ethylene in the ripening of these non-climacteric fruits? Journal of Experimental Botany 56, 2037–2046 15955790 10.1093/jxb/eri202

[CIT0060] Unwin RD Griffiths JR Whetton AD 2010 Simultaneous analysis of relative protein expression levels across multiple samples using iTRAQ isobaric tags with 2D nano LC-MS/M**S.** Nature Protocols 5, 1574–1582 10.1038/nprot.2010.123 21085123

[CIT0061] Waldron KW Parker M Smith AC 2003 Plant cell walls and food quality. Comprehensive Reviews in Food Science and Food Safety 2, 128–146 10.1111/j.1541-4337.2003.tb00019.x 33451229

[CIT0062] Wang AD Tan DM Tatsuki M Kasai A Li TZ Saito H Harada T 2009 Molecular mechanism of distinct ripening profiles in ‘Fuji’ apple fruit and its early maturing sports. Postharvest Biology and Technology 52, 38–43

[CIT0063] Wang Y Ji K Dai S 2013 The role of abscisic acid in regulating cucumber fruit development and ripening and its transcriptional regulation. Plant Physiology and Biochemistry 64, 70–79 23376370 10.1016/j.plaphy.2012.12.015

[CIT0064] Xu Q Chen LL Ruan XA 2013 The draft genome of sweet orange (*Citrus sinensis*). Nature Genetics 45, 59–66 23179022 10.1038/ng.2472

[CIT0065] Xu Q Yu KQ Zhu AD Ye JL Liu Q Zhang JC Deng XX 2009 Comparative transcripts profiling reveals new insight into molecular processes regulating lycopene accumulation in a sweet orange (*Citrus sinensis*) red-flesh mutant. BMC Genomics 10, 540 19922663 10.1186/1471-2164-10-540 PMC2784484

[CIT0066] Yu K Xu Q Da X Guo F Ding Y Deng X 2012 Transcriptome changes during fruit development and ripening of sweet orange (*Citrus sinensis*). BMC Genomics 13, 10 22230690 10.1186/1471-2164-13-10 PMC3267696

[CIT0067] Yun Z Jin S Ding Y Wang Z Gao H Pan Z Xu J Cheng Y Deng X 2012 Comparative transcriptomics and proteomics analysis of citrus fruit, to improve understanding of the effect of low temperature on maintaining fruit quality during lengthy post-harvest storage. Journal of Experimental Botany 63, 2873–2893 22323274 10.1093/jxb/err390 PMC3350911

[CIT0068] Zhang M Yuan B Leng P 2009 The role of ABA in triggering ethylene biosynthesis and ripening of tomato fruit. Journal of Experimental Botany 60, 1579–1588 19246595 10.1093/jxb/erp026 PMC2671613

[CIT0069] Zheng BB Wu XM Ge XX Deng XX Grosser JW Guo WW 2012 Comparative transcript profiling of a male sterile cybrid pummelo and its fertile type revealed altered gene expression related to flower development. PLoS One 7, e43758 22952758 10.1371/journal.pone.0043758 PMC3429507

[CIT0070] Zhu MM Dai SJ McClung S Yan XF Chen SX 2009 Functional differentiation of *Brassica napus* guard cells and mesophyll cells revealed by comparative proteomics. Molecular & Cellular Proteomics 8, 752–766 19106087 10.1074/mcp.M800343-MCP200 PMC2667361

