# Supplementary material for: An integrative analysis of the transcriptome and proteome of the pulp of a spontaneous late-ripening sweet orange mutant and its wild type improves our understanding of fruit ripening in citrus
Source: J Exp Bot. 2014 Mar 5;65(6):1651–71. doi: 10.1093/jxb/eru044 (PMC3967095; doi:10.1093/jxb/eru044)
Supplement: Supplementary Data [file supp_65_6_1651__index.html]

An integrative analysis of the transcriptome and proteome of the pulp of a spontaneous late-ripening sweet orange mutant and its wild type improves our understanding of fruit ripening in citrus — An integrative analysis of the transcriptome and proteome of the pulp of a spontaneous late-ripening sweet orange mutant and its wild type improves our understanding of fruit ripening in citrus — Supplementary Data 

# An integrative analysis of the transcriptome and proteome of the pulp of a spontaneous late-ripening sweet orange mutant and its wild type improves our understanding of fruit ripening in citrus

## Supplementary Data

Data files

**Files in this Data Supplement:**

- Supplementary Data - Supplementary Data
- Supplementary Data - Supplementary Data
- Supplementary Data - Supplementary Data
- Supplementary Data - Supplementary Data
- Supplementary Data - Supplementary Data
- Supplementary Data - Supplementary Data
- Supplementary Data - Supplementary Data
- Supplementary Data - Supplementary Data
- Supplementary Data - Supplementary Data
- Supplementary Data - Supplementary Data
- Supplementary Data - Supplementary Data
- Supplementary Data - Supplementary Data
