# Supplementary material for: An integrative analysis of the transcriptome and proteome of the pulp of a spontaneous late-ripening sweet orange mutant and its wild type improves our understanding of fruit ripening in citrus
Source: J Exp Bot. 2014 Mar 5;65(6):1651–71. doi: 10.1093/jxb/eru044 (PMC3967095; doi:10.1093/jxb/eru044)
Supplement: Supplementary Data [file supp_eru044_Supplementary_Table_S6._Several_important_KEGG_pathways_involved_in_MT_and_WT.docx]

**Supplementary Table S6.** Several important KEGG pathways involved in MT and WT. KEGG: Kyoto Encyclopedia of Genes and Genomes. MT: mutant type; WT: wild type; 170, 190 and 210 indicate the development stage 170, 190 and 210 DAF (days after flowering), respectively.

| KEGG Pathway | WT gene number | |  | MT gene number | | |
| --- | --- | --- | --- | --- | --- | --- |
|  | 190/170 | 210/190 |  | 190/170 | | 210/190 |
| Plant-pathogen interaction | 35 | 40 |  | 63 | | 31 |
| Plant hormone signal transduction | 34 | 40 |  | 56 | | 34 |
| Stilbenoid, diarylheptanoid and gingerol biosynthesis | 14 | 14 |  | 41 | | 15 |
| Microbial metabolism in diverse environments | 17 | 13 |  | 41 | | 15 |
| Phenylpropanoid biosynthesis | 18 | 19 |  | 31 | | 14 |
| Flavonoid biosynthesis | 9 | 10 |  | 29 | | 9 |
| Polycyclic aromatic hydrocarbon degradation | 7 | 9 |  | 28 | | 9 |
| Limonene and pinene degradation | 8 | 8 |  | 27 | | 9 |
| Starch and sucrose metabolism | 17 | 14 |  | 20 | | 10 |
| Protein processing in endoplasmic reticulum | 1 | 3 |  | 18 | | 2 |
| Apoptosis | 8 | 4 |  | 16 | | 5 |
| Diterpenoid biosynthesis | 3 | 3 |  | 14 | | 5 |
| Pentose and glucuronate interconversions | 9 | 6 |  | 13 | | 5 |
| Phenylalanine metabolism | 8 | 7 |  | 13 | | 8 |
| Flavone and flavonol biosynthesis | 2 | 4 |  | 12 | | 4 |
| RNA transport | 1 | 9 |  | 12 | | 5 |
| Ascorbate and aldarate metabolism | 6 | 3 |  | 7 | | 4 |
| Carotenoid biosynthesis | 8 | 4 |  | 7 | | 5 |
| Cysteine and methionine metabolism | 1 | 6 |  | 7 | | 6 |
| Spliceosome | 3 | 3 |  | | 7 | 3 |
