# Supplementary material for: An integrative analysis of the transcriptome and proteome of the pulp of a spontaneous late-ripening sweet orange mutant and its wild type improves our understanding of fruit ripening in citrus
Source: J Exp Bot. 2014 Mar 5;65(6):1651–71. doi: 10.1093/jxb/eru044 (PMC3967095; doi:10.1093/jxb/eru044)
Supplement: Supplementary Data [file supp_eru044_Supplementary_Results.doc]

**Supplementary Results**

*Transcriptome sequencing*

In total, six mRNA samples were sequenced from the fruit pulps of WT and MT, which were sampled at 170, 190 and 210 DAF. The average number of reads produced for each library was 7.29 million, and a total of 43,786,734 successful sequences were obtained (Table 1). After filtering, the number of clean reads per library ranged from 7.01 to 7.56 million. Sequence saturation analysis indicated that the growth rate of the detected genes tended towards saturation, with the number of reads increasing. When the number of reads reached 2.5 million, the growth rate of the detected transcripts became flattened (Fig. 1). We concluded that the libraries were all fully saturated and, hence, large enough for gene expression analysis. Of the total reads, more than 99% were defined as high quality (clean reads) by removing low-quality reads with ambiguous nucleotides and adaptor sequences (Table 1). The high-quality sequences were aligned to the recently released sweet orange (*C. sinensis* L. Osbeck) genome (http://citrus.hzau.edu.cn/orange/) (*Xu et a*l., 2013), which allowed for a mismatch in two bases. Of the clean reads, more than 79% were matched to a unique genomic location or multiple genomic locations (Table 1). Only the reads that uniquely mapped to the genes of the reference genome were used in the gene expression analysis of all of the libraries. In this study, 19,635, 19,638, 18,709, 19,265, 19,004 and 18,389 genes were identified in each successive stage of WT and MT fruit ripening respectively. (Table 1).

**Table 1.** Summary of the RNA-seq data collected from MT and WT at each of three selected fruit developmental stages. MT: mutant type; WT: wild type. DAF: days after flowering.

| **Category** |  | **170 DAF** |  | **190 DAF** |  | **210 DAF** |  |  |
| --- | --- | --- | --- | --- | --- | --- | --- | --- |
| Total Reads | WT | 7,609,910 |  | 7,405,129 |  | 7,237,114 |  | |
|  | MT | 7,306,357 |  | 7,174,154 |  | 7,054,070 |  | |
| Clean reads | WT | 7,564,255 | 99.40% | 7,367,467 | 99.49% | 7,198,939 | 99.47% | |
|  | MT | 7,267,115 | 99.46% | 7,137,039 | 99.48% | 7,018,085 | 99.49% | |
| unique match to genome | WT | 5,589,551 | 73.89% | 5,486,245 | 74.47% | 5,307,864 | 73.73% | |
|  | MT | 5,375,724 | 73.97% | 5,273,364 | 73.89% | 5,161,263 | 73.54% | |
| unique match to gene | WT | 5,093,325 | 67.33% | 4,981,626 | 67.62% | 4,789,013 | 66.52% | |
|  | MT | 4,930,237 | 67.84% | 4,791,309 | 67.13% | 4,670,199 | 66.55% | |
| Number of genes | WT | 19,635 |  | 19,638 |  | 18,709 |  |  |
|  | MT | 19,265 |  | 19,004 |  | 18,389 |  |  |


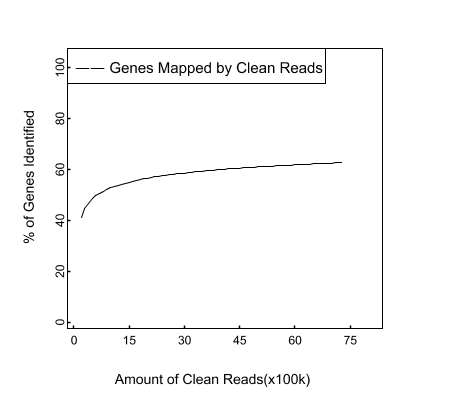

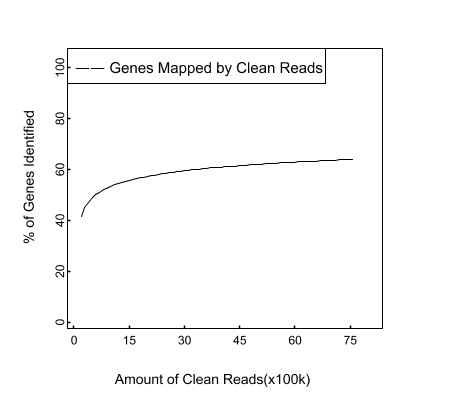


MT 170 DAF WT 170 DAF


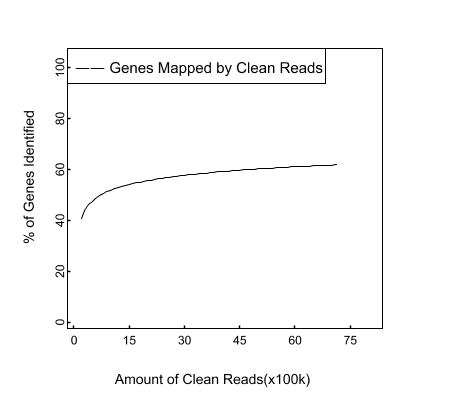

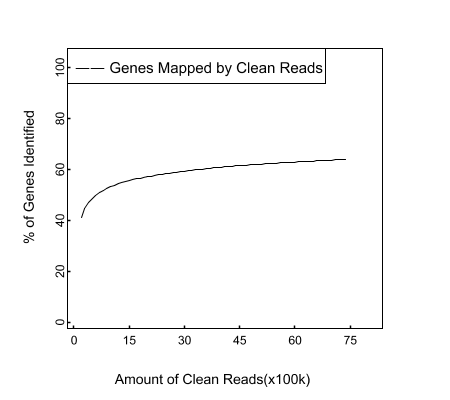


MT 190 DAF WT 190 DAF


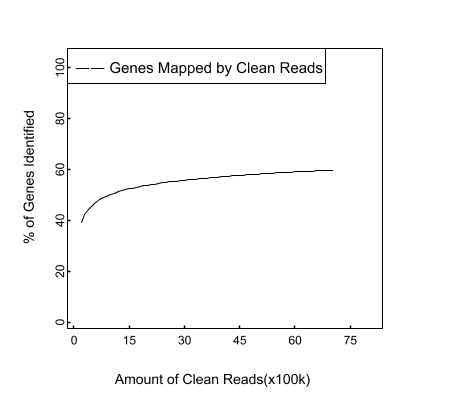

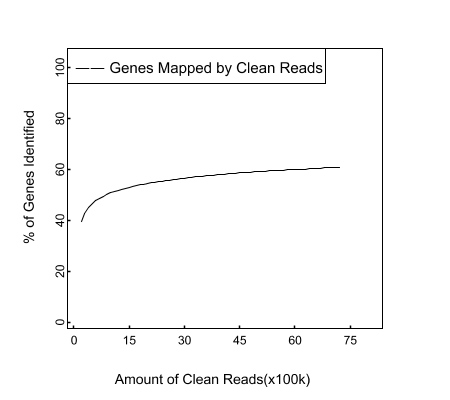


MT 210 DAF WT 210 DAF

**Fig. 1.** Saturation evaluation of the RNA-seq reads in the six libraries (MT and WT at three selected fruit developmental stages) against sequencing depth. As the sequencing depth was increased, the number of genes identified increased, but the number stabilized once the number of sequences reached 2.5 million. MT: mutant type; WT: wild type. MT 170 DAF: sampled from MT at 170 DAF (days after flowering).

**Xu Q, Chen LL, Ruan XA, Chen DJ, Zhu AD, Chen CL, Bertrand D, Jiao WB, Hao BH, Lyon MP, Chen JJ, Gao S, Xing F, Lan H, Chang JW, Ge XH, Lei Y, Hu Q, Miao Y, Wang L, Xiao SX, Biswas MK, Zeng WF, Guo F, Cao HB, Yang XM, Xu XW, Cheng YJ, Xu J, Liu JH, Luo OJ, Tang ZH, Guo WW, Kuang HH, Zhang HY, Roose ML, Nagarajan N, Deng XX, Ruan YJ**. 2013. The draft genome of sweet orange (Citrus sinensis). *Nature Genetics* **45**, 59-U92.
