# Supplementary material for: An integrative analysis of the transcriptome and proteome of the pulp of a spontaneous late-ripening sweet orange mutant and its wild type improves our understanding of fruit ripening in citrus
Source: J Exp Bot. 2014 Mar 5;65(6):1651–71. doi: 10.1093/jxb/eru044 (PMC3967095; doi:10.1093/jxb/eru044)
Supplement: Supplementary Data [file supp_eru044_Supplementary_Fig._S1._The_distribution_of_differential_proteins_in_COG_function_categories._.docx]

**Supplementary Fig. S1.** The distribution of differential proteins between MT and WT in COG function categories. COG: Cluster of Orthologous Groups of proteins. A: Posttranslational modification, protein turnover, chaperones; B: General function prediction only; C: Carbohydrate transport and metabolism; D: Energy production and conversion; E: Translation, ribosomal structure and biogenesis; F: Amino acid transport and metabolism; G: Replication, recombination and repair; H: Transcription; I: Signal transduction mechanisms; J: Secondary metabolites biosynthesis, transport and catabolism; K: Nucleotide transport and metabolism; L: Inorganic ion transport and metabolism; M: Function unknown; N: Cytoskeleton; O: Coenzyme transport and metabolism; P: Lipid transport and metabolism; Q: Intracellular trafficking, secretion, and vesicular transport; R: Cell wall/membrane/envelope biogenesis. DAF: days after flowering. MT: mutant type; WT: wild type.
